# Data-driven discovery of performance boundaries in robotic systems through extreme quantile estimation

**DOI:** 10.1371/journal.pone.0352196

**Published:** 2026-11-10

**Authors:** Hadi Jahanshahi, Zheng Hong Zhu

**Affiliations:** Department of Mechanical Engineering, York University, Toronto, Ontario, Canada; Montclair State University, UNITED STATES OF AMERICA

## Abstract

Characterizing the performance limits of physical systems from experimental data is essential for engineering decision-making, design optimization, and reliability analysis, yet conventional regression models estimate average behavior rather than the boundary of what is achievable. This paper develops a data-driven methodology for discovering nonlinear performance boundaries from experimental observations. The problem is formulated as extreme conditional quantile estimation. A nonparametric binned percentile estimator recovers low-quantile boundary points, and the Bayesian Information Criterion selects an interpretable closed-form law from a library of physically motivated families. Non-asymptotic guarantees are stated in the fixed-design regime the experiments occupy. The method is validated on two real robotic datasets. For UR10e trajectory tracking (30,600 observations), it discovers a saturation boundary $E=\text{7}.\text{7}\text{4}\left(\text{1}-e^{-\text{1}\text{3}.\text{2}\text{7}S}\right)$ mm ($R^{\text{2}}=\text{0}.\text{9}\text{6}\text{2}$; bootstrap 95% CI for $E_{\text{max}}$ of [7.05, 9.10] mm) and separates it from a steady-state envelope obeying the lag law $e=t_{\text{lag}}\cdot v$ with $t_{\text{lag}}=\text{6}\text{5}$ ms ($R^{\text{2}}=\text{0}.\text{9}\text{9}\text{7}$) once start-up transients are excluded. For planar pushing (40,378 pushes from the MIT MCube dataset), it discovers a power-law boundary with exponent α=2.02 (95% CI [1.88, 2.17]), indicating that the root-mean-square force fluctuation grows linearly with speed, consistent with momentum-transfer scaling at the breakdown of the quasi-static contact assumption. Family selection is stable across quantile levels and bootstrap resampling schemes, recovers the correct family in every synthetic replicate at the experimental configurations for the two family classes discovered here, and is confirmed by a prospectively specified experiment. Six of six extrapolation predictions at never-tested speeds landed within 1.2 mm, and a repeat of the protocol reproduced the per-episode 2nd percentiles to a mean absolute deviation of 0.039 mm. Compared with quantile regression, Gaussian process, support vector, and frontier-estimation baselines, the framework provides the tightest interpretable boundary estimates while approaching the target validity level, and it extrapolates where nonparametric envelopes cannot.

## 1. Introduction

In many physical systems, the key question is not what usually happens, but what can happen at best. A robot arm may track slow motions with little error, yet its accuracy inevitably declines as commanded speed increases. A pushing robot moves an object smoothly at low speed, while higher speeds produce large contact-force fluctuations and violate the quasi-static assumptions formalized by Lynch and Mason [1]. Engineering performance, in other words, is often constrained by hidden physical limits, and these limits are invisible to models built to predict typical behavior near the center of the data distribution [2,3].

Physical limits appear in data as the lower or upper edge of the observation cloud, which marks the best achievable performance at each operating condition or the onset of a regime where simplified physical assumptions fail. This study aims to discover such performance boundaries from experimental data. A performance boundary, unlike a trend line, encodes constraints imposed by system dynamics, control bandwidth, actuator limits, contact mechanics, or energy transfer. Once identified in closed form, it can support design, controller comparison, safety assessment, and operating-regime selection.

Existing tools address parts of this problem. Koenker and Bassett [4] introduced regression quantiles, later developed comprehensively by Koenker [5]; Meinshausen [6] extended conditional quantile estimation to random forests, and Takeuchi et al. [7] to kernel machines. These estimators, however, either presuppose a functional form or return curves without one, and Chernozhukov [8] documents the instability of quantile regression deep in the tails. A separate tradition estimates efficiency frontiers. Farrell [9] initiated the measurement of productive efficiency, Aigner, Lovell and Schmidt [10] formulated stochastic frontier analysis (SFA), and Charnes, Cooper and Rhodes [11] proposed data envelopment analysis (DEA) [12,13], yet these methods were built for production economics rather than physical feasibility. Gaussian processes (GPs) [2] and support vector regression (SVR) [3] can supply prediction bounds, but such bounds quantify statistical uncertainty instead of the limit of what a system can do.

To address this gap, this study develops an interpretable framework for discovering performance boundaries using extreme quantile learning. The method partitions the input range, estimates low-quantile boundary points in each region, and fits physically meaningful candidate families, including saturation, power-law, logarithmic, square-root, and linear models. The boundary is selected by the Bayesian Information Criterion (BIC), whose interpretation as approximate Bayes-factor evidence follows Kass and Raftery [14], and the estimator comes with finite-sample guarantees stated in the fixed-design regime the experiments occupy. The output is a closed-form equation suitable for interpretation, prediction, and engineering decision support.

The framework is evaluated on two robotic datasets and one prospectively specified validation experiment. The first dataset involves trajectory tracking with a UR10e manipulator collected in the authors’ laboratory, where tracking error is analyzed as a function of commanded speed across three trajectory geometries. The second uses the planar pushing benchmark of Yu et al. [15] for contact-force analysis; pushing is a demanding test case because, as Bauza and Rodriguez [16] and Ajay et al. [17] show, contact dynamics resist modeling once speed increases and the quasi-static assumption fails. Together the cases probe actuator and controller limits on one hand and contact-dynamics limits on the other, and the discovered boundaries are subsequently tested prospectively at never-fitted speeds and geometries.

The main contributions of this paper are as follows:

1. An extreme conditional quantile formulation is introduced for performance-boundary discovery in physical systems.
2. An interpretable boundary-discovery framework is developed using binned percentile estimation and BIC-based model selection.
3. Consistency and convergence properties of the estimator are derived under standard assumptions.
4. The method is validated on two real robotic datasets, recovering distinct physical-limit behaviors.
5. Comparisons with quantile regression (QR), GPs, and SVR show improved balance among tightness, validity, and interpretability.

## 2. Related work

The proposed framework is related to four research areas: QR, frontier estimation, machine-learning-based envelope estimation, and data-driven model discovery in robotics. This section reviews these areas and clarifies the gap addressed by this study.

### 2.1. Quantile regression

QR estimates a conditional quantile $Q_{\tau }(Y|X=x)$ as a function of x [18–23]. The standard formulation parametrizes the quantile as $\beta_{\tau }^{⊤}\phi (x)$ for a feature map ϕ and minimizes the asymmetric pinball loss. Polynomial, spline, and kernel bases extend the linear formulation [24–27].

However, for performance boundary discovery, the relevant quantile is extreme (τ→0), where pinball-loss estimators become numerically unstable and often require specialized asymptotic treatment [8,28]. Moreover, the functional form of the boundary is usually chosen in advance by the analyst. This limits its usefulness when the physical boundary may follow an unknown saturation, logarithmic, polynomial, or power-law pattern. The framework proposed in this paper departs from QR by combining a nonparametric per-bin quantile estimator, which bypasses the extreme-quantile instability, with model selection over a library of physically motivated parametric families.

A rapidly developing branch of this literature addresses conditional extreme quantiles directly. Tang, Chen, Li and Wang [29] survey the field. Because the target quantile lies at or beyond the edge of the data, essentially all methods share a two-stage strategy. They first estimate at an intermediate quantile level, where observations remain, and then extrapolate outward using extreme-value theory, with the conditional extreme value index governing tail heaviness as a function of the covariates. The recent wave they synthesize replaces classical parametric machinery with semiparametric QR, tree ensembles, and deep learning for one or both stages, with applications spanning insurance, hydrology, and finance. The present framework operates in a complementary regime. The operating level τ = 0.02 is moderately extreme, chosen so that direct within-bin estimation remains feasible (Section 6.7), and the extrapolation machinery reviewed in [29] and developed by Chernozhukov [8] is the natural route to the τ → 0 limit discussed in Section 3 and Section 7.

### 2.2. Frontier estimation

Frontier estimation has been widely studied in production economics and efficiency analysis [10,12]. SFA assumes a parametric production function and decomposes deviations into inefficiency and noise components. DEA [11,13] estimates the frontier nonparametrically as the upper envelope of the convex hull of observed inputs and outputs. The free-disposal hull (FDH) of Deprins, Simar and Tulkens [30] dispenses with convexity and estimates the frontier by the minimum of dominating observations, which makes it maximally tight but sensitive to a single extreme point, a fragility that Section 6.10 confirms empirically. Cazals, Florens and Simar [31] introduced the order-m expected-minimum frontier, and Aragon, Daouia and Thomas-Agnan [32] the order-α conditional-quantile frontier; Daouia and Simar [33] extended these ideas to robust frontier estimation.

Although these methods provide strong statistical foundations, they are formulated for upper production frontiers. Their machinery transfers to a lower envelope after a sign change, but their assumptions are not always suitable for physical robotic systems, where the object of interest is a lower envelope of a cost-like outcome. Production frontiers are commonly motivated by economic efficiency and often assume monotonicity, convexity, or specific returns-to-scale behavior. In contrast, physical-system boundaries may arise from actuator limits, controller bandwidth, contact mechanics, or energy-transfer effects, and their shape may be saturating, sublinear, superlinear, or power-law-like. The proposed framework adapts the conditional-quantile foundation to physical systems where the boundary shape is itself unknown and must be discovered from data.

### 2.3. Machine learning for envelope estimation

Several machine-learning methods can be adapted to approximate data envelopes. GPs [34–36] and SVR [37–39] fit a conditional mean and a confidence band; the lower edge of the band serves as a heuristic boundary. These methods do not target the lower envelope directly. They minimize prediction error to all data points, and their boundary heuristic is determined by the noise model instead of by the physical limit. Conformal prediction [40–43] provides distribution-free prediction intervals but again does not target the lower envelope. One-class support vector machines [44–47] estimate the support of a distribution instead of its conditional quantile.

These methods are useful for uncertainty estimation, anomaly detection, and support estimation, but they do not directly target a physical performance boundary. Gaussian-process and conformal intervals describe predictive uncertainty, and not the best achievable physical performance. Support vector methods and one-class classifiers can approximate data margins, but the resulting boundaries are often difficult to interpret physically. By contrast, the proposed framework directly targets the conditional lower quantile while also discovering the parametric form.

### 2.4. Data-driven model discovery in robotics

Data-driven model discovery has become increasingly important in robotics and physical-system modeling, leading to many principled approaches to discovering closed-form models from data [48–51]. Sparse identification of nonlinear dynamics (SINDy) [48] discovers governing equations as sparse linear combinations of basis functions; symbolic regression [52,53] searches the space of expressions directly. These methods usually model system dynamics or average input-output behavior, while the proposed framework for performance-boundary discovery is complementary, in that it estimates the limiting curve separating feasible from infeasible performance. This is especially relevant in robotics, where operating limits can be more important than average behavior.

For planar pushing in particular, the MIT MCube benchmark [15] has been used extensively to evaluate planar contact models. Bauza and Rodriguez [16] showed that contact-force prediction is highly variable at high speeds. Ajay et al. [17] learned residual dynamics correcting an analytic quasi-static prior. However, none of these works estimate the explicit boundary of force-variance versus speed. The proposed framework provides a novel quantitative characterization of where the quasi-static assumption breaks down on the MCube dataset.

Boundary discovery also connects to a broader program of data-driven discovery of physical laws. Schmidt and Lipson [49] demonstrated that free-form natural laws can be distilled from experimental data by symbolic regression, and Brunton, Proctor and Kutz [48] made discovery scalable through sparse regression over a candidate function library. Yuan et al. [54] extended this lineage to cyber-physical systems. Their inference of hybrid dynamical equations (IHYDE) framework identifies, from data alone, how many subsystems a hybrid system contains, the governing equations of each, and the transition logic between them, without prior specification of any of the three. That literature discovers the governing dynamics of typical behavior; the present framework discovers envelope laws of extremal behavior. The two are complementary in practice. A discovered dynamics model predicts trajectories, while a discovered performance boundary certifies the limits of what those trajectories can achieve. Both share the commitment to closed-form, physically interpretable output that distinguishes discovery from black-box regression.

### 2.5. Research gap

Existing methods address parts of the problem but rarely combine extreme-boundary estimation, data-driven model selection, closed-form interpretability, and validation on real robotic systems. The proposed framework fills this gap by converting experimental observations into explicit knowledge about physical performance limits.

Table 1 summarizes how the proposed framework compares with the closest existing approaches across five attributes. Among the methods compared here, the proposed framework is the only one designed to target the conditional lower quantile, discover the parametric form from data, and return an interpretable closed-form equation at the same time. It is also the only one that combines these properties with validation on real robotic systems and an explicit physical interpretation of the fitted parameters.

**Table 1:** Positioning of the proposed framework against closest existing approaches.

Approach	Targets lower envelope	Discovers form	Closed-form output	Robotic validation	Physical interpretation
Linear QR	yes	no	yes	limited	no
Polynomial QR	yes	no	yes	limited	no
GP (μ−2σ)	no	no	no	yes	no
SVR	no	no	no	yes	no
DEA/ SFA frontiers	yes	partial	yes	no	limited
Order-m and order-α frontiers	yes	no	no	no	no
SINDy/ symbolic regression	no	yes	yes	yes	yes
**Proposed framework**	**yes**	**yes**	**yes**	**yes**	**yes**

## 3. Problem formulation

This section defines the performance boundary as a conditional quantile, states the assumptions under which it is identifiable, defines the candidate parametric families, defines the binned percentile estimator, and specifies the performance metrics used in evaluation.

### 3.1. Definitions

**Definition 1** (Quantile Boundary). For a system with input X∈X⊆R and output Y∈R jointly distributed as $P_{X,Y}$, and for a quantile level τ∈(0,1), the quantile boundary at level τ is


$$\begin{array}{c} f_{\tau }^{*}(x)=Q_{Y|X}(\tau |x)=\text{inf}\{y\in \text{R}:F_{Y|X}(y|x)\geq \tau \} \end{array}$$ (1)

where $F_{Y|X}$ is the conditional cumulative distribution function of Y given X=x.

**Definition 2** (Essential Infimum Boundary). The *essential infimum boundary* is the limit


$$\begin{array}{c} f_{\text{0}}^{*}(x)=\underset{\tau \to {\text{0}}^{+}}{\text{lim}}f_{\tau }^{*}(x) \end{array}$$ (2)

This limit, when it exists and is finite, is the supremum of the values y for which Pr(Y≤y∣X=x)=0.

The quantile boundary $f_{\tau }^{*}(x)$ at small but positive τ is the primary estimand of this work, and it is referred as the *performance boundary at level* τ. It is the level below which performance better than y occurs in at most a fraction τ of operating instances at condition x. Under Assumption 2 below, $f_{\tau }^{*}(x)$ decreases monotonically to $f_{\text{0}}^{*}(x)$ as τ→0, so the performance boundary is a conservative, statistically estimable surrogate for the essential-infimum boundary. Every empirical claim in this paper concerns $f_{\tau }^{*}$ at the operating level τ=0.02; the choice of τ and the sensitivity of all conclusions to it are analyzed in Section 6.

**Remark 1** (When the limit is degenerate). For some outcome variables the essential infimum is uninformative even though performance boundaries at moderate extreme levels are physically meaningful. Instantaneous tracking error is an example. Whenever the commanded velocity reverses direction, the signed error passes near zero, so arbitrarily small instantaneous errors occur transiently at every commanded speed and $f_{\text{0}}^{*}(x)\approx \text{0}$ uniformly. The level-τ boundary, by contrast, characterizes the sustained best-case regime and remains informative. This is why $f_{\tau }^{*}$, not $f_{\text{0}}^{*}$, is the estimand of this framework, and why the dependence of discovered boundaries on τ is reported explicitly. Extrapolation of $f_{\tau }^{*}$ toward τ→0 using extreme-value theory [8,29] offers a principled bridge to the idealized limit and is discussed as future work in Section 7.

**Definition 3** (Boundary Estimation Problem). Given observations $\{(X_{i},Y_{i}){\}}_{i=\text{1}}^{n}\sim P_{X,Y}\;$ and a quantile level τ, the boundary estimation problem is to construct an estimator ${\hat{f}}_{\tau }:X\to R$ such that ${\hat{f}}_{\tau }(x)$ approximates $f_{\tau }^{*}(x)$ uniformly over X.

**Definition 4** (Parametric Boundary Families). A parametric boundary family is a set F={f(·;θ):θ∈Θ} of candidate boundary functions indexed by a parameter θ in a compact parameter space $\Theta \subseteq R^{p}$. Five families are considered in this paper:


$$\begin{array}{c} \text{linear}:f(x;a,k)=a+kx \end{array}$$ (3)

$$\begin{array}{c} \text{square root}:f(x;a,k)=a+k\sqrt{x} \end{array}$$ (4)

$$\begin{array}{c} \text{logarithmic}:f(x;a,k)=a+k\text{log}(\text{1}+x) \end{array}$$ (5)

$$\begin{array}{c} \text{power law}:f(x;a,k,\alpha )=a+kx^{\alpha } \end{array}$$ (6)

$$\begin{array}{c} \text{saturation}:f(x;a,k,E_{\text{max}})=a+E_{\text{max}}(\text{1}-e^{-kx}) \end{array}$$ (7)

**Definition 5** (Binned Percentile Estimator). Let $B_{\text{1}},\ldots ,B_{B}$ be a partition of X into B disjoint bins, and let ${\bar{x}}_{b}$ denote the centroid of bin $B_{b}$. For each bin, let $S_{b}=\{Y_{i}:X_{i}\in B_{b}\}$ and $n_{b}=\left|S_{b}\right|$. The binned percentile estimator of the quantile boundary is the piecewise-constant function defined by


$$\begin{array}{c} {\hat{q}}_{\tau ,b}=\text{empirical }\tau -\text{quantile of }S_{b},b=\text{1},\ldots ,B \end{array}$$ (8)

together with a parametric refinement obtained by least-squares fitting of a family F:


$$\begin{array}{c} \hat{\theta }=\text{arg}\underset{\theta \in \Theta }{\text{min}}\sum_{b=\text{1}}^{B}({\hat{q}}_{\tau ,b}-f({\bar{x}}_{b};\theta ){)}^{\text{2}} \end{array}$$ (9)

The full estimator is ${\hat{f}}_{\tau }(x)=f(x;\hat{\theta })$.

### 3.2. Assumptions

The framework relies on five assumptions.

**Assumption 1** (Monotone boundary). The function $f_{\tau }^{*}(x)$ is monotonically non-decreasing in x over the operating range X.

**Assumption 2** (Regular lower tail at level τ). For each bin b, the conditional distribution $F_{b}=F_{Y|X}\left(\cdot |x_{b}\right)$ admits a density $p_{b}$ that is continuous and bounded below, $p_{b}(y)\geq c_{b}>\text{0}$ for all $y\in \left[f_{\tau }^{*}\left(x_{b}\right)-\delta ,\;f_{\tau }^{*}\left(x_{b}\right)+\delta \right]$, for some δ>0 and constants $c_{b}>\text{0}$; write $c={\text{min}}_{b}c_{b}$.

**Assumption 3** (Smooth parametric boundary). The function $f_{\tau }^{*}(\cdot )$ admits a parametric representation $f_{\tau }^{*}(x)=f\left(x;\theta^{*}\right)$ for some $\theta^{*}$ in a compact set $\Theta \subseteq R^{p}$, where θ↦f(x;θ) is twice continuously differentiable for each x.

**Assumption 4** (Identifiability on the design). The map $\theta \mapsto \left(f\left(x_{\text{1}};\theta \right),\ldots ,f\left(x_{B};\theta \right)\right)$ is injective on Θ, and at $\theta^{*}$ the B×p gradient matrix G with rows $\nabla_{\theta }f{\left(x_{b};\theta^{*}\right)}^{⊤}$ has full column rank p (hence B≥p).

**Assumption 5** (Sampling). Conditional on the design, the observations within bin b are independent draws from $F_{b}$, independent across bins.

Assumption 1 reflects the physical intuition that more demanding operating conditions cannot yield better best-case performance; its empirical status is tested directly on the boundary points in Section 6, where an apparent violation at the highest tracking speed is traced to the interpolation behavior of the sample quantile rather than to the system. The assumption underpins the interpretation of the boundary as a performance limit; it is not invoked in the proofs of Theorems 1–3. Assumption 2 differs from the usual support-boundary conditions of frontier analysis in a way that matters here. Positivity of the density is required only in a neighborhood of the τ-quantile, where estimation actually occurs, and no assumption about the lower endpoint of the support is needed at all. Assumption 4 is stated on the finite design because that is the form that is checkable in practice; full column rank of G at the design speeds is verified numerically for the selected family on each design in Section 6. Assumption 5 is the idealization appropriate for independently collected trials (one observation per physical push, as in the pushing dataset); Section 4.5 develops the modification required when observations arrive in correlated batches, as in the tracking dataset.

### 3.3. Performance metrics

The framework is evaluated by four metrics, defined consistently across all methods compared in this paper:

- *Coefficient of determination* $R^{\text{2}}$, computed against the per-bin τ-quantile points (the boundary points themselves), measuring how well the fitted parametric model captures the observed boundary.
- *Validity*, the fraction of raw observations $\left\{(X_{i},Y_{i})\right\}$ that lie above or on the predicted boundary. For an estimator that correctly targets the τ-quantile, validity should equal 1−τ≈98%.
- *Tightness*, the mean absolute error between the predicted boundary and the per-bin τ-quantile values, in the units of Y.
- *Monotonicity*, a binary indicator of whether the predicted boundary is non-decreasing on a dense grid covering X.

A method that targets the boundary correctly will have validity close to 1−τ. Validity substantially below this target indicates a method that cuts through the data and does not represent any meaningful boundary. Validity substantially above the target indicates a method that sits well below the boundary, achieving high coverage by being conservative rather than accurate. The framework is designed to target the boundary itself, and not to maximize validity.

## 4. Theoretical properties

This section establishes the statistical properties of the binned percentile estimator and the BIC selection step. All results are stated in the sampling regime the experiments actually occupy. The condition variable takes values on a finite design $x_{\text{1}}<\cdots <x_{B}$ of commanded speeds, B is a design constant fixed by the experiment, and asymptotics are driven by the per-bin sample sizes $n_{b}$, with $n_{\text{min}}={\text{min}}_{b}n_{b}$. A growing-design regime (B→∞) is retained only as a corollary. The backbone of all downstream results is a non-asymptotic concentration bound that holds simultaneously for every finite $\left(B,n_{\text{1}},\ldots ,n_{B}\right)$.

### 4.1. A non-asymptotic uniform bound on the per-bin quantiles

**Lemma 1** (Uniform per-bin quantile concentration). Under Assumptions 2 and 5, for every ε∈(0,δ],


$$\begin{array}{c} \text{Pr}\left(\underset{b}{\text{max}}\left|{\hat{q}}_{\tau ,b}-f_{\tau }^{*}\left(x_{b}\right)\right|>\varepsilon \right)\;\leq \;\text{2}\sum_{b=\text{1}}^{B}e^{-\text{2}n_{b}c_{b}^{\text{2}}\varepsilon^{\text{2}}}\;\leq \;\text{2}B\;e^{-\text{2}n_{\text{min}}c^{\text{2}}\varepsilon^{\text{2}}}. \end{array}$$ (10)

Equivalently, for any η∈(0,1), with probability at least 1−η, simultaneously for all b,


$$\begin{array}{c} \left|{\hat{q}}_{\tau ,b}-f_{\tau }^{*}\left(x_{b}\right)\right|\;\leq \;\varepsilon_{b}(\eta )\;=\;\sqrt{\frac{\text{log}(\text{2}B/\eta )}{\text{2}\;n_{b}\;c_{b}^{\;\text{2}}}}. \end{array}$$ (11)

**Proof.** Fix b and ε≤δ. If ${\hat{q}}_{\tau ,b}>f_{\tau }^{*}\left(x_{b}\right)+\varepsilon$, then ${\hat{F}}_{b}\left(f_{\tau }^{*}\left(x_{b}\right)+\varepsilon \right)<\tau$, while Assumption 2 gives $F_{b}\left(f_{\tau }^{*}\left(x_{b}\right)+\varepsilon \right)\geq \tau +c_{b}\varepsilon$; hence ${\text{sup}}_{y}\left|{\hat{F}}_{b}(y)-F_{b}(y)\right|>c_{b}\varepsilon$. The symmetric argument applies when ${\hat{q}}_{\tau ,b}<f_{\tau }^{*}\left(x_{b}\right)-\varepsilon$. By the Dvoretzky–Kiefer–Wolfowitz inequality with Massart’s constant [55], $\text{Pr}\left({\text{sup}}_{y}\left|{\hat{F}}_{b}(y)-F_{b}(y)\right|>c_{b}\varepsilon \right)\leq \text{2}e^{-\text{2}n_{b}c_{b}^{\text{2}}\varepsilon^{\text{2}}}$. A union bound over b=1,…,B yields the first inequality of (10); bounding each $n_{b}$ by $n_{\text{min}}$ and $c_{b}$ by c yields the second. Inverting the tail bound bin by bin, with η/B allocated to each bin, gives (11).◻

Unlike an asymptotic statement, Lemma 1 is valid at every finite configuration, including the experimental ones, and its constants are estimable. Substituting kernel plug-in estimates of the tail densities $c_{b}$ (Section 6) with η=0.05 certifies, under the independence idealization of Assumption 5 and for the tracking dataset (B=8, $n_{\text{min}}=\text{1}\text{0}\text{8}\text{9}$), simultaneous envelopes ranging from ±0.06 mm at the lowest commanded speed to envelopes of several millimeters at the highest speeds, where the lower tail is flat, the data carries less information about the boundary, and Section 6.6 shows the bound ceases to be informative. Two further computations bear on the same bins. The per-bin asymptotic standard deviation of the empirical quantile, denoted $\sigma_{b}=\sqrt{\tau (\text{1}-\tau )}\;/\;\left(p_{b}\left(f_{\tau }^{*}\left(x_{b}\right)\right)\sqrt{n_{b}}\right)$ evaluates to 1.26 mm at the 0.30 m/s bin, so a normal 95% interval there spans about 4.9 mm against the 2.3 mm width of the cluster bootstrap reported in Section 6. The three computations differ in how conservative they are, and they agree in identifying the same bins as the least informative, namely the bins where the lower tail is flattest and each episode contributes the fewest timesteps. The envelopes of (11) are therefore presented as finite-sample certificates that are informative at the low-speed bins and, as Section 6.6 quantifies, not applicable at the highest speeds, where the certified neighborhood would leave the region in which Assumption 2 holds. The bootstrap intervals are the calibrated uncertainty used for inference throughout.

**Corollary 1** (Asymptotic regimes). From (11): (i) for fixed B, ${\text{max}}_{b}\left|{\hat{q}}_{\tau ,b}-f_{\tau }^{*}\left(x_{b}\right)\right|\to \text{0}$ in probability as $n_{\text{min}}\to \infty$, at rate $O_{P}\left(n_{\text{min}}^{-\text{1}/\text{2}}\right)$; (ii) if instead $B=B_{n}\to \infty$, uniform consistency holds whenever $\text{log}B_{n}/n_{\text{min}}\to \text{0}$. Regime (i) is the one occupied by both experiments in this paper and is the regime in which all subsequent results are stated.

### 4.2. Pointwise consistency

**Theorem 1** (Pointwise consistency). Under Assumptions 2 and 5, for each design point $x_{b}$, ${\hat{q}}_{\tau ,b}\to f_{\tau }^{*}\left(x_{b}\right)$ in probability as $n_{b}\to \infty$. If X is continuous and binned, the same conclusion holds for interior points x of bin $B_{b}$ provided additionally the bin width tends to zero and $f_{\tau }^{*}$ is continuous at x.

**Proof.** The first claim is immediate from Lemma 1 applied to the single bin b. For the binned case, decompose ${\hat{q}}_{\tau ,b}-f_{\tau }^{*}(x)=\left({\hat{q}}_{\tau ,b}-f_{\tau ,b}\right)+\left(f_{\tau ,b}-f_{\tau }^{*}(x)\right)$, where $f_{\tau ,b}$ is the τ-quantile of the within-bin mixture distribution. The first term vanishes in probability by Lemma 1 applied with $F_{b}$ the mixture. For the second, continuity of $f_{\tau }^{*}$ and shrinking bin width imply that the mixture’s lower tail is sandwiched between those of the extremal conditional distributions in the bin, whose τ-quantiles both converge to $f_{\tau }^{*}(x)$.

### 4.3. Parameter estimation at a fixed design

Let $\hat{\theta }=\text{arg}{\text{min}}_{\theta \in \Theta }\sum_{b=\text{1}}^{B}({\hat{q}}_{\tau ,b}-f\left(x_{b};\theta \right){)}^{\text{2}}$ denote the least-squares stage of the pipeline, and let $\theta^{*}$ be as in Assumption 3.

**Theorem 2** (Finite-sample deviation and asymptotic normality at a fixed design). Under Assumptions 2–5:

(a) (Finite-sample bound.) Let $M={\text{sup}}_{\theta \in \Theta }{\text{max}}_{b}\left|f_{\tau }^{*}\left(x_{b}\right)-f\left(x_{b};\theta \right)\right|$, let κ denote the smallest eigenvalue of $G^{⊤}G$, and let $r_{\text{0}}>\text{0}$ and $q_{\text{0}}>\text{0}$ be the quadratic-growth radius and the outside-gap of the population criterion defined in S1 Appendix. On the event of Lemma 1 with tolerance $\varepsilon ={\text{max}}_{b}\varepsilon_{b}(\eta )$, if $B\varepsilon (\text{2}M+\varepsilon )<q_{\text{0}}/\text{2}$, then


$$\begin{array}{c} ∥\hat{\theta }-\theta^{*}∥\;\leq \;\text{2}\;\sqrt{\frac{B\;\varepsilon \;(\text{2}M+\varepsilon )}{\kappa }}. \end{array}$$ (12)

(b) (Asymptotic normality.) If $n_{b}/n\to w_{b}\in (\text{0},\text{1})$ as n→∞ with B fixed, then


$$\begin{array}{c} \sqrt{n_{\text{min}}}\;\left(\hat{\theta }-\theta^{*}\right)\;\Rightarrow \;N\left(\text{0},\;{\left(G^{⊤}G\right)}^{-\text{1}}G^{⊤}S\;G\;{\left(G^{⊤}G\right)}^{-\text{1}}\right), \end{array}$$ (13)

where S is the diagonal matrix with entries


$$\begin{array}{c} S_{bb}\;=\;\frac{n_{\text{min}}}{n_{b}}\;s_{b}^{\text{2}},\;\;s_{b}^{\text{2}}\;=\;\frac{\tau (\text{1}-\tau )}{p_{b}(f_{\tau }^{*}\left(x_{b}\right){)}^{\text{2}}}. \end{array}$$ (14)

The proof is given in S1 Appendix. Part (a) is a non-asymptotic certificate whose constants are computable at the experimental configuration. Here rank(G)=p=3 holds for the selected family at the design speeds of both datasets, with $\kappa /B=\text{1}.\text{7}\times {\text{1}\text{0}}^{-\text{3}}$ (tracking) and $\text{9}.\text{3}\times {\text{1}\text{0}}^{-\text{3}}$ (pushing). Part (b) recovers the familiar $\sqrt{n_{\text{min}}}$ rate of nonlinear least squares at a fixed design [56], driven entirely by per-bin sample sizes; the number of design points B enters only through the fixed matrices G and S. Unlike growing-design analyses, this result does not require B→∞ with ${\text{min}}_{b}n_{b}\cdot B^{-\text{2}}\to \infty$, a regime the experiments, with B∈{8,10}, do not occupy.

### 4.4. Model Selection: Consistency and Misspecification

With candidate families $F_{\text{1}},\ldots ,F_{K}$ having $p_{\text{1}},\ldots ,p_{K}$ parameters, define for each k the population projection error on the design,


$$\begin{array}{c} D_{k}\;=\;\underset{\theta \in \Theta_{k}}{\text{min}}\sum_{b=\text{1}}^{B}(f_{\tau }^{*}\left(x_{b}\right)-f_{k}\left(x_{b};\theta \right){)}^{\text{2}}, \end{array}$$ (15)

so that $D_{k}=\text{0}$ exactly when family k contains the boundary on the design. The framework selects $\hat{k}=\text{arg}{\text{min}}_{k}{\text{BIC}}_{k}$ with


$$\begin{array}{c} {\text{BIC}}_{k}\;=\;B\text{log}\left(\frac{{\text{RSS}}_{k}}{B}\right)+p_{k}\text{log}B, \end{array}$$ (16)

where ${\text{RSS}}_{k}$ is the residual sum of squares of family k on the B bin points. The interpretation of BIC differences follows the conventional thresholds of Kass and Raftery [14].

**Theorem 3** (Selection consistency at a fixed design). Suppose exactly one candidate family satisfies Assumptions 3 and 4 on the design, so that its projection error vanishes while every other family has a strictly positive projection error. Then under Assumptions 2–5, the probability that BIC selects that family tends to one as the per-bin sample size grows with B fixed.

**Remark 2** (Nested families). The library is partly nested, since the linear and square-root families are the power law with its exponent fixed at one and at one half. If a boundary is contained in two nested families at once, both residual sums of squares vanish at the same rate, their ratio converges to a non-degenerate random limit, and the parameter-count penalty is a constant at fixed B. BIC then prefers the smaller family with a probability that tends to a limit strictly below one rather than to one, and consistent selection within such a pair would require a growing design rather than the fixed design used here. A simulation reported in Section 6.9 places that limit near three quarters. This case does not arise in either dataset, because no family nested within the selected one has zero projection error; the BIC margins reported in Sections 6.2 and 6.3, together with an exponent interval that excludes both 1 and 0.5, are the evidence for that.

**Proposition 1** (Behavior under misspecification). Suppose that no family contains $f_{\tau }^{*}$ on the design, and let $\tilde{k}=\text{arg}{\text{min}}_{k}D_{k}$. If this minimizer is unique and no competing family offsets a larger projection error with a smaller parameter count, a condition that holds automatically for competitors with at least as many parameters and that is verified numerically for the configurations reported here, then the probability of selecting the projection-optimal family tends to one as the per-bin sample size grows, and the fitted parameter converges in probability to the pseudo-true projection parameter of that family. If several families attain the same minimal projection error, the one with the fewest parameters is selected with probability tending to one.

Proofs are given in S1 Appendix; the key display is that for the correct (or projection-optimal) family the first BIC term diverges,


$$\begin{array}{c} B\text{log}\left(\frac{{\text{RSS}}_{k^{*}}}{B}\right)\;\to \;-\infty \;\text{in probability as }n_{\text{min}}\to \infty , \end{array}$$ (17)

while for every other family it is bounded in probability, and the parameter-count penalties are constants at fixed B. Theorem 3 and Proposition 1 are verified empirically in the synthetic study of Section 6. Selection accuracy reaches 100% at the experimental $\left(B,n_{\text{min}}\right)$ for saturation- and power-type ground truths. For a ground truth outside the library, the projection-optimal family is selected in all replicates and the fitted curve tracks the true τ-quantile curve closely. Selection degrades exactly where the theory predicts, for near-degenerate family pairs over short ranges at small B, a regime that small reported ΔBIC margins flag in practice.

### 4.5. Dependent Data: Clusters and effective sample size

Assumption 5 fails when observations arrive in trajectories or batches. Within-episode autocorrelation makes the informative sample size the number of independent episodes, and not the number of recorded timesteps, so nominal-n formulas do not apply. All reported intervals for the tracking dataset are therefore episode-level cluster-bootstrap confidence intervals for the boundary of the task family, conditional on the design; the pushing intervals come from a push-level bootstrap, since each push is an independent trial. They are the calibrated uncertainty used for inference throughout. Two modifications cover this case. First, on the theory side, if bin b contains $m_{b}$ independent episodes contributing equal numbers of observations, Lemma 1 holds with $n_{b}$ replaced by an effective size proportional to $m_{b}$. Changing any single episode changes the pooled empirical distribution function by at most $\text{1}/m_{b}$ in supremum norm, so McDiarmid’s inequality [57] applied to the supremum deviation from its mean yields the same exponential bound with the effective size in place of the nominal one, up to the centering term that Section A.4 of S1 Appendix states explicitly, and the quantile conversion of Lemma 1 proceeds unchanged. For the tracking dataset $m_{b}=\text{9}$, so nominal-n formulas are not applicable; at the flattest bins the plug-in normal approximation is in fact the wider of the two, because the tail-density estimate entering it is small and unstable. Second, on the inference side, all reported uncertainty for the tracking dataset is therefore computed by an episode-level cluster bootstrap that resamples, with replacement, the nine episodes recorded at each commanded speed, with a design-stratified variant reported in Section 6.6 as a robustness check, never from nominal-n formulas. The pushing dataset, with one observation per physical push, satisfies Assumption 5 directly, and a shape-level cluster bootstrap is reported there as a conservative robustness check (Section 6).

## 5. Methodology

The full algorithmic pipeline consists of four stages: data collection, binned percentile estimation, parametric fitting, and model selection by BIC. Fig 1 illustrates these stages on real UR10e data; Algorithm 1 gives the formal specification.

**Fig 1:**
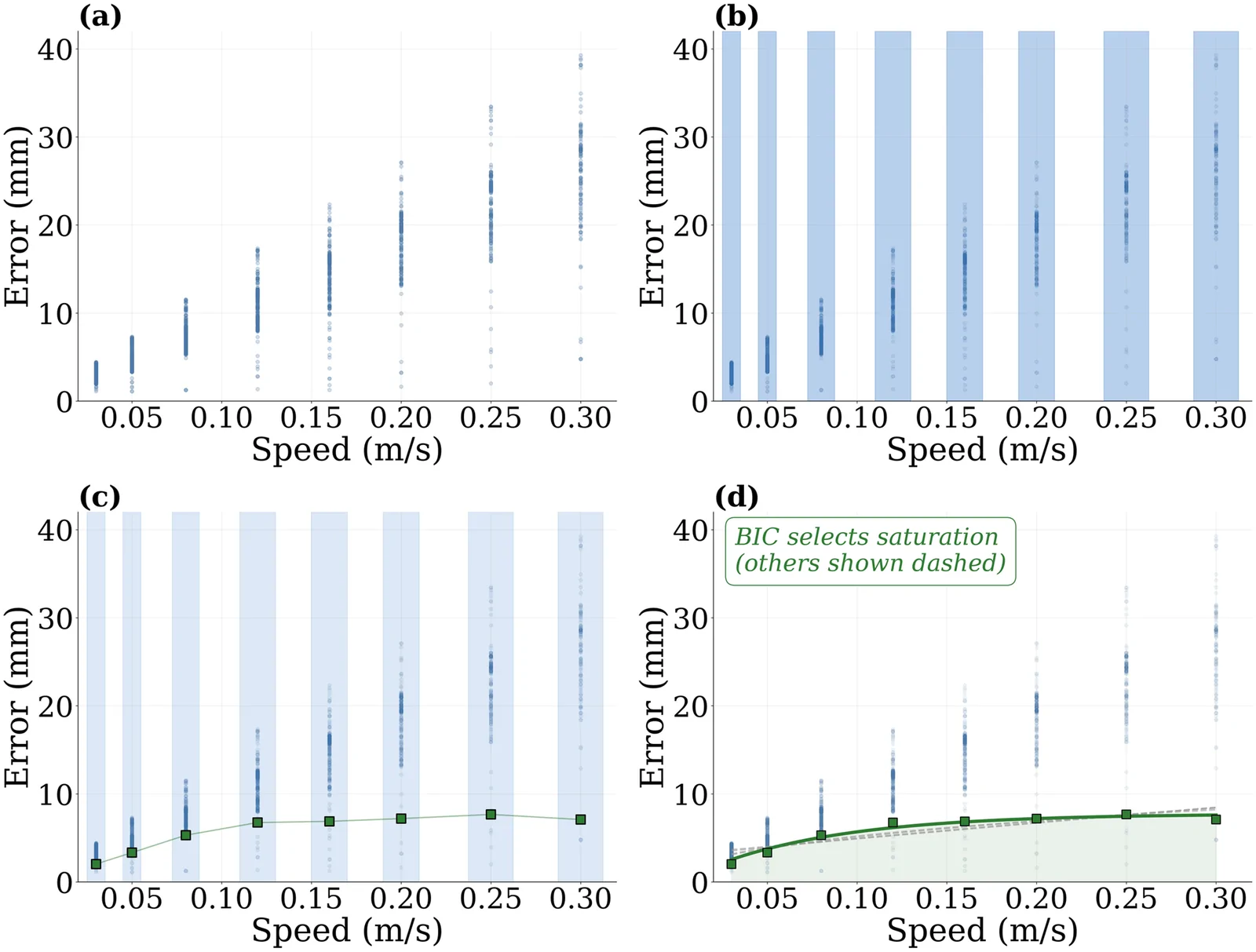
Four-stage boundary-discovery pipeline of the framework on UR10e robot tracking data.

**Algorithm 1:** Performance boundary discovery

**Require:** Observations ${\left\{(X_{i},Y_{i})\right\}}_{i=\text{1}}^{n}$, quantile level τ, candidate families$F_{\text{1}},\ldots ,F_{K}$

**Ensure:** Boundary function ${\hat{f}}_{\tau }$, selected family index $\hat{k}$

1. Determine bin grid $B_{\text{1}},\ldots ,B_{B}$ from the structure of X

2. **For** b=1,…,B **do**

3. $S_{b}\leftarrow \{Y_{i}:X_{i}\in B_{b}\}$

4. ${\hat{q}}_{\tau ,b}\leftarrow$empirical τ-quantile of$S_{b}$

5. ${\bar{x}}_{b}\leftarrow$centroid of$B_{b}$

6. **End for**

7. **For** k=1,…,K **do**

8. ${\hat{\theta }}_{k}\leftarrow \text{arg}{\text{min}}_{\theta }\sum_{b=\text{1}}^{B}({\hat{q}}_{\tau ,b}-f_{k}({\bar{x}}_{b};\theta ){)}^{\text{2}}$

9. ${\text{RSS}}_{k}\leftarrow \sum_{b}({\hat{q}}_{\tau ,b}-f_{k}({\bar{x}}_{b};{\hat{\theta }}_{k}){)}^{\text{2}}$

10. ${\text{BIC}}_{k}\leftarrow B\text{log}({\text{RSS}}_{k}/B)+p_{k}\text{log}B$

11. **End for**

12. $\hat{k}\leftarrow \text{arg}{\text{min}}_{k}{\text{BIC}}_{k}$

13. **Return** ${\hat{f}}_{\tau }(\cdot )=f_{\hat{k}}(\cdot ;{\hat{\theta }}_{\hat{k}})$,$\hat{k}$

### 5.1. Bin construction

For datasets with discrete commanded values of X (such as the eight target speeds in the robot tracking experiment or the ten pushing speeds in the MCube dataset), the bins are taken to coincide with these commanded values. Each bin $B_{b}$ is the set of observations whose nominal commanded X equals the b-th level. For datasets with continuous X, equal-width bins are used.

### 5.2. Per-bin quantile estimation

Within each bin, the empirical τ-quantile ${\hat{q}}_{\tau ,b}$ is computed from the $n_{b}$ observations. For τ=0.02 and per-bin sample sizes ranging from 1,000–11,000 (Section 6), the empirical 2nd percentile is well-defined and stable. The boundary point assigned to bin b is the pair $\left({\bar{x}}_{b},{\hat{q}}_{\tau ,b}\right)$.

### 5.3. Parametric fitting

The candidate families are those listed in Definition 4. The linear family is the conventional baseline. The square-root and logarithmic families capture sub-linear growth typical of diffusive processes. The power law captures super-linear growth typical of energy- or momentum-dominated phenomena. The saturation family captures systems whose boundary asymptotes to a finite ceiling. Each family is fitted to the B boundary points $\left({\bar{x}}_{b},{\hat{q}}_{\tau ,b}\right)$ by nonlinear least squares with non-negativity constraints on physically meaningful parameters.

### 5.4. Model selection

After fitting all K families, BIC is computed on the B bin points and the family with the lowest BIC is selected. Reporting the full set of fitted $R^{\text{2}}$ and BIC values exposes the selection rationale instead of presenting a single chosen family without justification. Section 6 reports both the selected family and the Δ BIC margin to all alternatives.

## 6. Experimental validation

The framework is validated on two distinct robotic domains, trajectory tracking on a UR10e arm (collected by the authors) and planar pushing on the publicly available MIT MCube dataset [15]. The two domains exhibit opposite physical regimes, namely a transient-limited error envelope in the first and quasi-static breakdown in the second. The validation establishes that the framework recovers the correct functional form in each domain and quantifies its advantage over standard baselines.

### 6.1. Datasets

The robot tracking dataset consists of 72 trajectory executions on a UR10e six-degree-of-freedom (6-DOF) arm at eight commanded target speeds in {0.03,0.05,0.08,0.12,0.16,0.20,0.25,0.30} m/s. The nine executions per speed follow a factorial design of three trajectory geometries (square, circle, figure-eight) times three repetitions, so that the discovered boundary is supported by three distinct curvature profiles instead of a single path shape. Each execution produces timestep-level observations of instantaneous tracking error at 125 Hz. Removing transitions with near-zero commanded velocity (60 of 30,660 transitions, 0.2%) leaves 30,600 observations, with per-speed counts from 11,022 at 0.03 m/s down to 1,089 at 0.30 m/s. Each observation is treated as an (X,Y) pair, where X is the commanded target speed of the parent trajectory and Y is the instantaneous tracking error. Repetitions are near-deterministic. Within each geometry-speed cell, the three repetitions correlate at 0.92–0.998 and their per-episode 2nd percentiles agree to within 0.02 mm in the median case and 0.12 mm at worst, so the effective replication unit is the episode, as formalized in Section 4.5 and reflected in all uncertainty computations below.

The pushing dataset is the public MIT MCube benchmark [15]. The subset analyzed here comprises all zero-acceleration pushes on the acrylonitrile butadiene styrene (ABS) surface. Of 73,494 hierarchical data format v5 (HDF5) files scanned, 40,786 contain a valid, complete force/torque trace with a parseable commanded speed, spanning eleven object shapes at ten commanded speeds in {10,…,500} mm/s. For each push, the output Y is the variance of the contact force magnitude over the push duration, so each push contributes exactly one observation. A 99th-percentile outlier filter removes 408 pushes (1.0%), leaving 40,378, with 3,776–4,083 pushes per speed. Because each push is an independent physical trial, Assumption 5 holds directly; a conservative shape-level cluster analysis is reported in Section 6.6.

### 6.2. Robot trajectory tracking

Fig 2 compares the five candidate parametric families fitted to the eight per-target 2nd-percentile boundary points. The saturation family achieves $R^{\text{2}}=\text{0}.\text{9}\text{6}\text{2}$, capturing the plateau in achievable tracking error at high speeds; alternatives fail to capture the saturation, with $R^{\text{2}}$ ranging from 0.700 for the linear family to 0.837 for the power law. The fitted saturation parameters are $E_{\text{max}}=\text{7}.\text{7}\text{4}$mm, k=13.27 s/m, and intercept negligibly close to zero, yielding the discovered boundary

**Fig 2:**
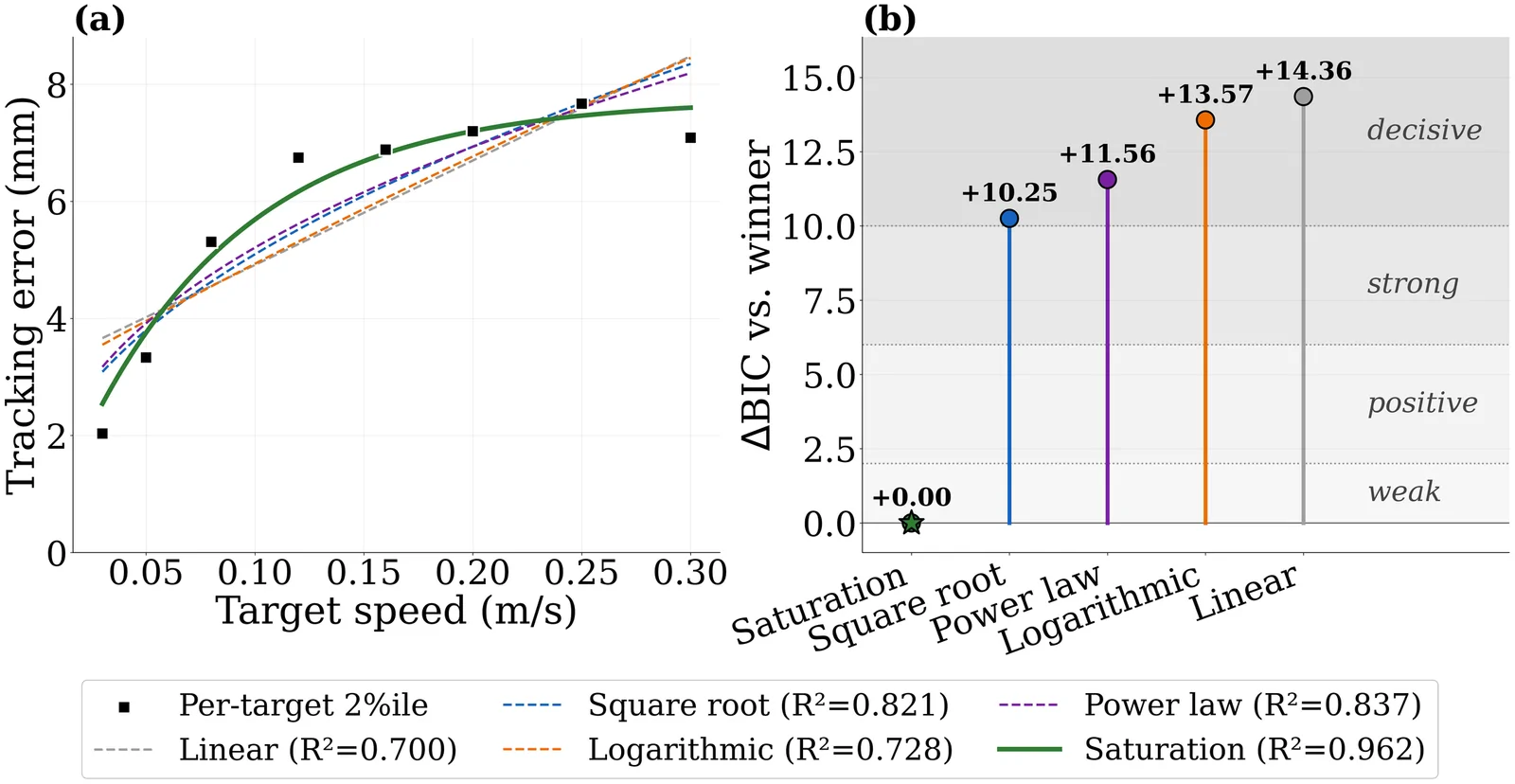
Candidate parametric families and BIC evidence for the robot tracking boundary. (a) Fitted families on the boundary points. (b) BIC evidence against the alternatives.


$$\begin{array}{c} E(S)=\text{7}.\text{74}(\text{1}-e^{-\text{13}.\text{27}S})\text{mm} \end{array}$$ (18)

The right panel of Fig 2 reports the ΔBIC margin between the selected family and each alternative. The smallest margin is to the square-root family at ΔBIC=10.25, which exceeds the conventional threshold for very strong evidence. The other alternatives lie further into the very strong zone. The selection of saturation over the alternatives is therefore strongly supported by the data.

The validity of the saturation fit is 92.1%, below the target of 98% corresponding to τ=0.02. This shortfall reflects the fact that smoothing eight noisy boundary points with a three-parameter parametric model unavoidably trades a small fraction of validity for substantially better fit fidelity. The tightness of the fit is 0.317 mm, meaning the predicted boundary lies on average within one-third of a millimeter of each observed bin’s 2nd percentile.

In (18) the commanded speed S is in m/s and the boundary E in mm, so $E_{\text{max}}$ = 7.74 mm and the rate constant k = 13.27 has units of s/m. The interpretation of this plateau requires care, and Section 6.5 examines it directly. The discovered saturation is a genuine, reproducible, and, as Section 6.11 shows, prospectively predictive property of the recorded process. Its physical origin, however, lies in the acceleration-limited start-up transient of each execution instead of in sustained tracking. At the highest speeds an episode contains only 103–131 timesteps, depending on geometry, so the 2nd percentile falls on the first few control cycles, before the steady-state lag has developed. Once these transients are excluded, the sustained best-case error follows a different and equally interpretable law (Section 6.5). Statistically, the fit is supported by an adjusted $R^{\text{2}}$ of 0.946 (residual degrees of freedom 5), a full-rank gradient matrix at the design speeds (rank G=3; Assumption 4 verified), and an episode-bootstrap 95% confidence interval (CI) for $E_{max}$ of [7.05,9.10] mm (Section 6.6).

### 6.3. Planar pushing

Fig 3 repeats the analysis for the pushing dataset. The power-law family achieves $R^{\text{2}}=\text{0}.\text{9}\text{9}\text{7}$ on the ten boundary points; alternatives produce $R^{\text{2}}$ ranging from 0.560 for the logarithmic family to 0.873 for the linear and saturation families. The fitted power-law parameters are $k=\text{5}.\text{3}\text{6}\times {\text{1}\text{0}}^{-\text{6}}$, α=2.02, and intercept a=0.012 N², yielding the discovered boundary

**Fig 3:**
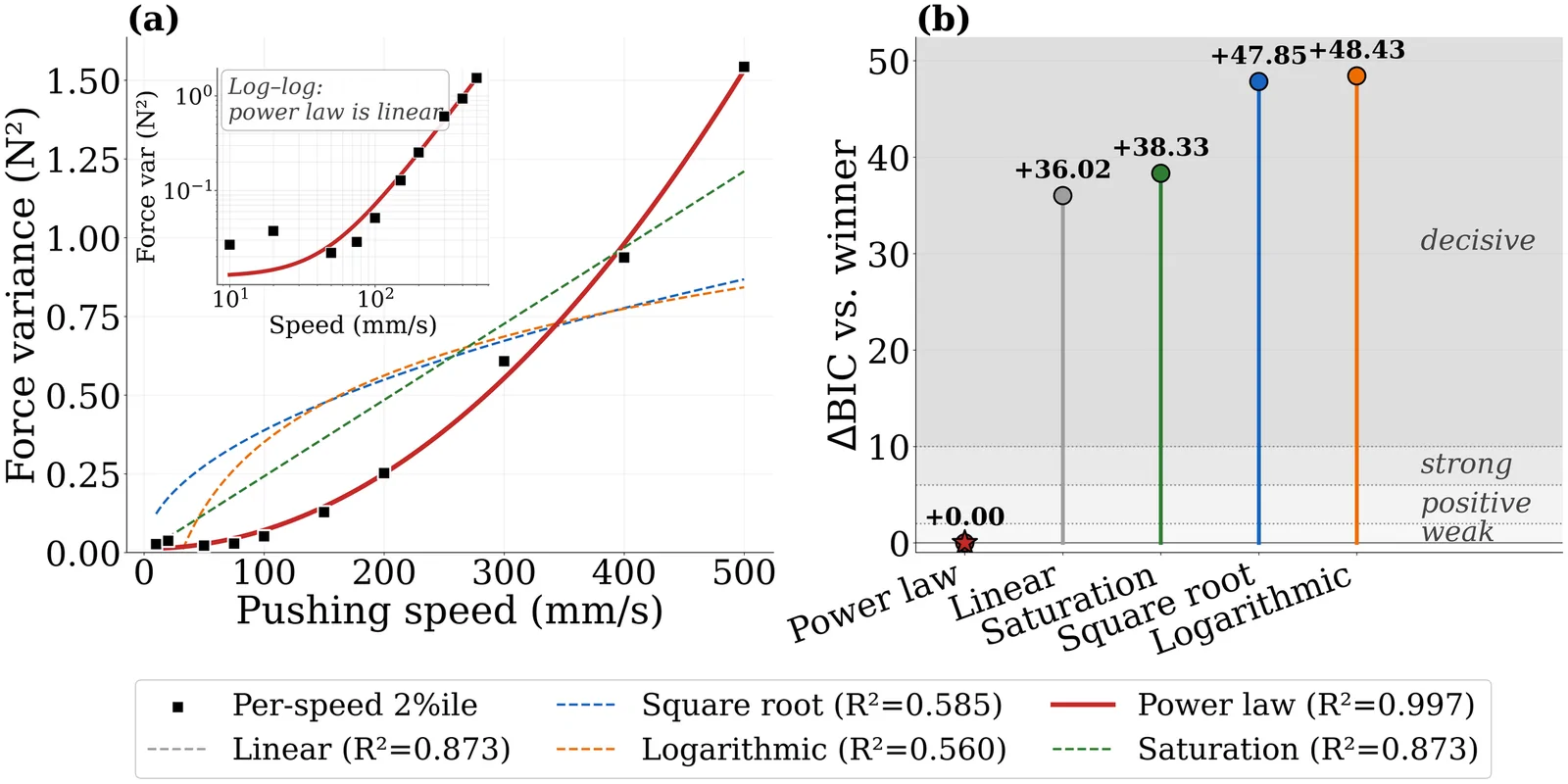
Candidate parametric families and BIC evidence for the planar pushing boundary. (a) Fitted families on the boundary points. (b) BIC evidence against the alternatives.


$$\begin{array}{c} E(S)=\text{0}.\text{012}+\text{5}.\text{36}\times {\text{10}}^{-\text{6}}S^{\text{2}.\text{02}}{\text{N}}^{\text{2}} \end{array}$$ (19)

The inset in Fig 3 shows the same fit on log-log axes, where the power-law manifests as a straight line and visually confirms the scaling relationship.

The fitted saturation family is degenerate on this dataset, operating in its linear regime, so its curve coincides with the linear fit in Fig 3a, and their BIC difference of 2.31 is essentially the parameter-count penalty log B = 2.30; the low-speed flattening visible in the inset reflects the additive floor a = 0.012 N². The fit is supported by an adjusted $R^{\text{2}}$ of 0.996 (residual degrees of freedom 7), a full-rank gradient matrix at the ten design speeds, and a push-level bootstrap 95% CI for the exponent of α∈[1.88,2.17], which contains the value α=2 (Section 6.6).

The ΔBIC margins exceed 36 for all alternatives, far into the very strong evidence zone. The validity of the power-law fit is 97.99%, essentially at the 98% target. The tightness is 0.0205 N², comparable to the smallest boundary values and about 75 times smaller than the largest. The fit is therefore both highly accurate and tightly aligned with the empirical boundary points.

The exponent α≈2.02 admits an immediate physical interpretation. Force fluctuations during a push are dominated by impact and stick-slip events. Each such event transfers momentum at a rate proportional to the sliding speed, so the amplitude of the fluctuating contact force grows linearly with speed, and its variance, a second moment, grows with the square of speed. An exponent of two is therefore the signature of momentum-transfer-dominated fluctuations, whereas fluctuations scaling with dynamic pressure would give an exponent near four and purely quasi-static friction an exponent near zero. The discovered exponent α≈2 is thus consistent with the breakdown of the quasi-static assumption that underlies most analytic pushing models [1], and quantifies the regime in which speed-driven momentum transfer begins to overwhelm the friction-dominated quasi-static regime.

### 6.4. Comparison with baselines

The framework is compared against three standard methods on consistent metrics. QR uses degree-2 polynomial features at τ=0.02. GP regression uses a radial basis function (RBF) kernel with the lower-bound heuristic $\hat{\mu }-\text{2}\hat{\sigma }$. SVR uses an RBF kernel with the lower edge of the ϵ-tube as the boundary heuristic. Table 2 reports all four methods on both datasets. The proposed framework achieves the highest $R^{\text{2}}$ on both datasets (0.962 and 0.997), the tightest fit on both datasets (0.317 mm and 0.0205 N²), and either approaches or matches the validity target. The QR baseline meets the validity target on tracking (98.0%) but only by sitting systematically below the boundary points. Its $R^{\text{2}}$ is 0.696, indicating that the polynomial fit captures the boundary’s shape much less accurately than saturation. The GP baseline on tracking is highly conservative (98.9% validity) but has $R^{\text{2}}=-\text{7}.\text{1}\text{9}$, meaning the predicted boundary is worse than predicting the mean of the boundary points; in other words, the GP heuristic sits well below the boundary instead of targeting it. The SVR baseline fails the validity criterion on both datasets (69.1% and 59.4%), confirming that an ϵ-tube around the conditional mean is not aligned with the lower envelope.

**Table 2:** Baseline comparison on both datasets.

Dataset	Method	$R^{2}$	Validity	Tightness	Monotonic
Robot tracking	Proposed (saturation)	**0.962**	92.1%*	**0.317 mm**	yes
	QR (poly-2)	0.696	**98.0%**	0.824 mm	yes
	GP (μ−2σ)	−7.19	98.9%†	4.475 mm	yes
	SVR (lower edge)	−29.93	69.1%*	8.416 mm	yes
Object pushing	Proposed (power)	**0.997**	**98.0%**	**0.0205 N²**	yes
	QR (poly-2)	0.062	90.7%*	0.415 N²	no
	GP (μ−2σ)	−4.25	97.6%	0.544 N²	yes
	SVR (lower edge)	−130.85	59.4%*	4.362 N²	no

Bold: best value per dataset (for validity, closest to the 98% target).

† Validity exceeds target due to overly conservative boundary estimate.

* Validity below 95%, well under the targeted level 1 − τ = 98% (for the proposed method on tracking, see Section 6.4).

The validity criterion of Section 3.3 separates the baselines sharply. GP on tracking shows the conservative failure mode, with validity above target at 98.9% but

$R^{\text{2}}$of −7.19. SVR on both datasets shows the opposite one, cutting through the data at 69.1% and 59.4%. The proposed framework on tracking lies below the target (92.1%) because the parametric saturation model cannot pass through all eight boundary points exactly, which is the unavoidable bias-variance tradeoff of parametric smoothing. On pushing it lies essentially exactly at the target (98.0%), the more favorable scenario where the power-law form happens to align well with the underlying data-generating process.

### 6.5. Two-regime structure of the tracking boundary

The tracking data contain two envelopes, and the framework separates them cleanly. Fig 4 presents both envelopes together with their fitted laws. The boundary reported in Section 6.2 is computed on episodes as recorded; because each episode begins with the servo accelerating from rest toward a target that departs at the commanded speed, the smallest errors in every episode form a short deterministic ramp. At low speeds, episodes are long (up to 1,331 timesteps) and the 2nd percentile clears this ramp; at high speeds (103–131 timesteps per episode at 0.30 m/s, depending on geometry) the 2nd percentile falls inside it. Excluding the first 10% of each episode’s timesteps and recomputing the boundary yields a different and simpler law. The steady-state 2nd-percentile error grows as $e=t_{lag}v$ with $t_{lag}=\text{6}\text{5}$ ms and $R^{\text{2}}=\text{0}.\text{9}\text{9}\text{7}$ with no free intercept, a direct measurement of the closed-loop tracking lag time constant, consistent in magnitude with the servo controller lookahead horizon of 100 ms. Because one millisecond multiplied by one meter per second equals one millimeter, $t_{lag}$ expressed in milliseconds gives e directly in millimeters. The lag law is a one-parameter model motivated by the physics rather than a member of the five-family library, whose members all carry an intercept; BIC applied to the library selects a non-saturating family for the trimmed boundary, and the lag law is reported because it fits the same points with no free intercept. The result is insensitive to the trim fraction (5%, 10%, and 20% trims are indistinguishable; Fig 4b), and the same family is selected at every trim level. Both envelopes were checked at never-fitted speeds (Section 6.11); the recorded-process envelope through the prospectively specified predictions, and the steady-state envelope through a post hoc comparison that was not part of that set. At 0.35 and 0.40 m/s the recorded-process envelope remains in the predicted asymptote regime (8.28 and 8.90 mm), while the steady-state envelope reaches 20.9 and 22.9 mm against lag-law predictions of 22.6 and 25.9 mm, so the law is conservative by 8–13 percent at these speeds. The two-regime structure carries a general methodological lesson that Remark 1 anticipated. The definition of what constitutes an observation is part of the estimand, and performance boundaries at extreme quantiles can transition between physical regimes exactly where the number of observations below the quantile, $n_{b}\tau$ becomes comparable to the number of timesteps in the start-up transient.

**Fig 4:**
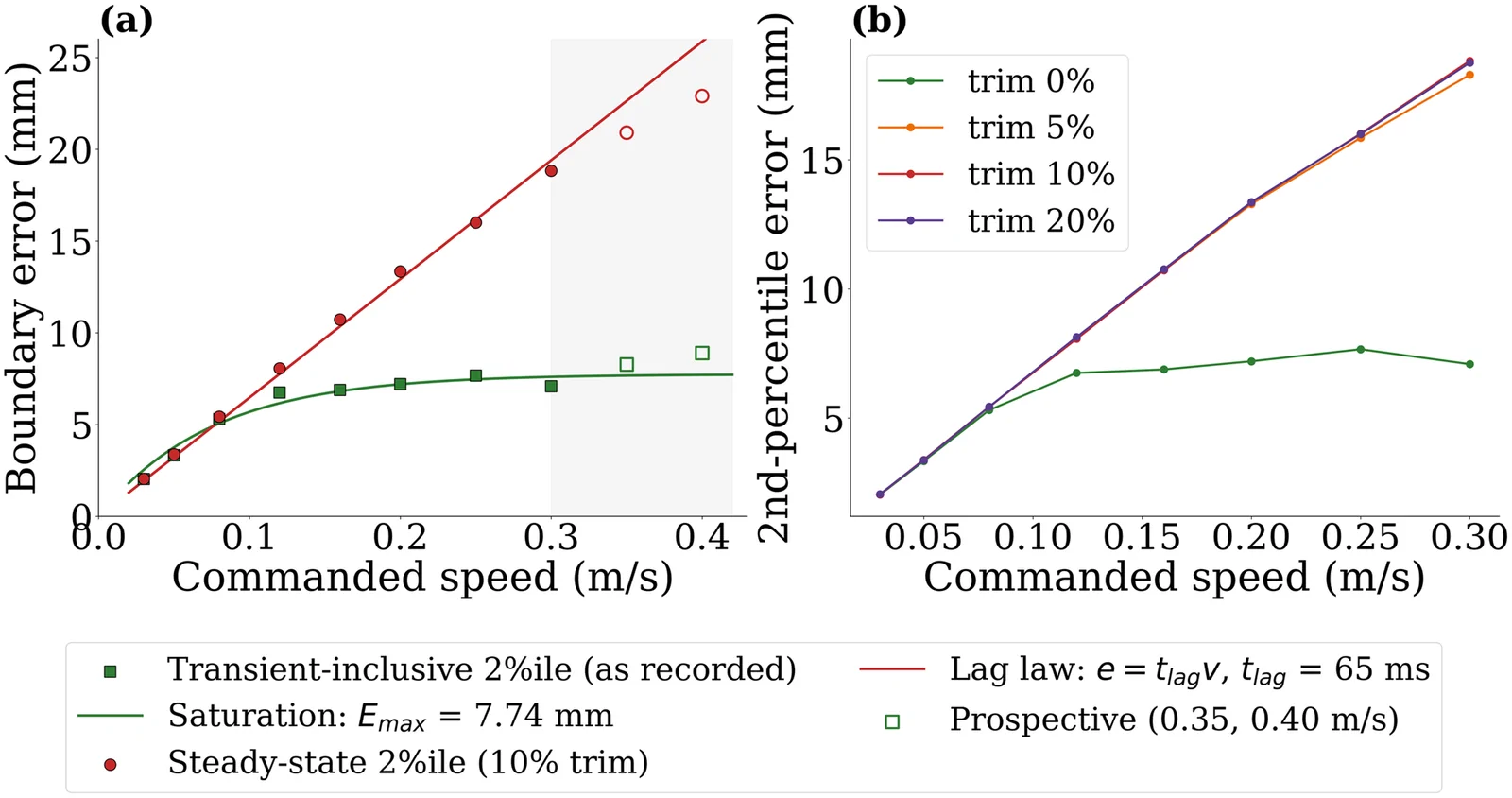
Two-regime structure of the tracking boundary. (a) Transient-inclusive and steady-state envelopes with their fitted laws. (b) Robustness of the steady-state boundary to the trim fraction.

### 6.6. Uncertainty quantification

Fig 5 summarizes the uncertainty analyses. All tracking uncertainty is computed by an episode-level bootstrap, resampling the nine episodes within each commanded speed with replacement and re-running the entire pipeline (boundary points, all five fits, BIC selection) in each of 1,500 replicates. The saturation family is selected in 94% of replicates (the runner-up, square root, takes the remainder, consistent with its + 10.25 margin being the smallest in Fig 2), and in 100% of replicates under a design-stratified variant that resamples the three repetitions within each geometry-speed cell. The 95% CI for $E_{max}$ is [7.05,9.10] mm. Per-bin boundary-point intervals are tight at low speeds and widen to 2.1–2.4 mm at the highest speeds. The same bins carry the widest finite-sample certificates of Section 4, and the asymptotic per-bin standard deviation σ_b of Section 4.1, namely 1.26 mm at 0.30 m/s, gives a normal 95% interval of about 4.9 mm, so the computations differ in conservatism while localizing the uncertainty in the same bins (per-bin values and certificates in S1 Table). The discovery is also insensitive to the choice of sample-quantile estimator. Repeating the analysis with the type-8 and Harrell-Davis quantile estimators leaves the saturation family selected, $E_{\text{max}}$ at 7.59 and 7.74 mm against 7.74 mm, the rate constant within 2.2%, and the Harrell-Davis episode bootstrap gives an $E_{\text{max}}$ interval of [7.14, 8.42] mm that is contained in the reported one. The tail densities entering those certificates are Gaussian kernel density estimates evaluated at the per-bin 2nd percentile, and they range from 0.26 per mm at the lowest commanded speed to 0.0034 per mm at the highest, which is why the certificate widens from 0.06 mm at the lowest commanded speed to about 15 mm at the highest. At that bin the certified neighborhood would extend below zero error, where the density vanishes and Assumption 2 no longer holds, so the certificate is informative at low speeds and vacuous at the highest ones. That is precisely the regime in which the bootstrap intervals, rather than the worst-case bound, carry the information. The design-stratified bootstrap reproduces the decrease at 0.30 m/s in every replicate, so it is not sampling noise. The small decrease relative to 0.25 m/s (−0.58 mm, 7.5% of $E_{max}$) is reproducible under re-execution rather than sampling noise. It is, however, sensitive to the quantile estimator: recomputing every boundary point with the smoothed Harrell-Davis estimator turns the decrease into an increase of 0.29 mm, so the apparent violation reflects the interpolation behavior of the sample quantile at the bin with the fewest samples per episode rather than a physical property of the system. It also disappears in the steady-state envelope of Section 6.5.

**Fig 5:**
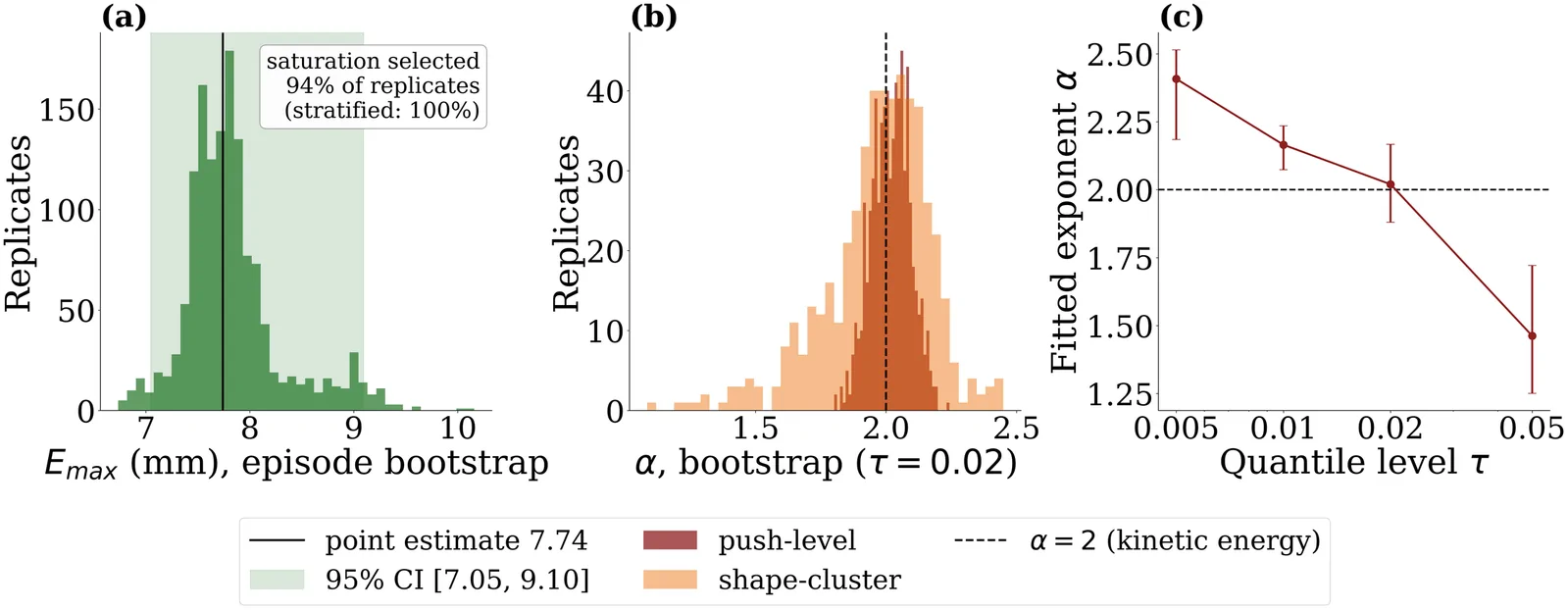
Uncertainty quantification and sensitivity to the quantile level. (a) Bootstrap distribution of the tracking asymptote $E_{max}$. (b) Bootstrap distributions of the pushing exponent α. (c) Dependence of α on the quantile level τ.

For pushing, a push-level bootstrap (each push an independent trial) selects the power law in every replicate and gives α∈[1.88,2.17]; a conservative shape-cluster bootstrap over the eleven object shapes also selects the power law in every replicate and widens the interval to [1.46,2.30]. Both intervals contain the momentum-transfer value α=2 (Fig 5b).

### 6.7. Sensitivity to the quantile level

Table 3 repeats the discovery at τ∈{0.005,0.01,0.02,0.05,0.10}. Family selection is stable across the extreme range on both datasets. Saturation wins for tracking and the power law wins for pushing at every τ≤0.05. Only at τ=0.10, where a quantile is no longer extreme, does the winner change, and in both cases by a statistical tie (ΔBIC of 0.04 and 0.16 to the previous winner). The operating level τ=0.02 is the smallest level that keeps at least ~20 observations below the quantile in the thinnest bin (21 for tracking, 75 for pushing). At τ=0.005 only five tail observations support the thinnest tracking bin and the fit degrades ($R^{\text{2}}=\text{0}.\text{7}\text{4}$), while at τ≥0.05 validity falls below its target of 1 − τ (90.5% against a 95% target at τ = 0.05), and at the largest level tested the quantile is no longer extreme. The pushing exponent varies systematically with τ (Table 3; Fig 5c): α decreases from 2.41 at τ=0.005 to 1.46 at τ=0.05, with the intervals at the extreme levels not overlapping, so the drift is real rather than noise. The summary is threefold. The qualitative discovery (a convex power law, α>1) is invariant across all extreme levels. The deepest estimable quantiles give α≥2, indicating that the extreme lower envelope is at least quadratic. The exponent of the τ→0 limit is not identifiable at fixed τ without extreme-value extrapolation [8,29], which is left to future work. The τ-dependence itself implies that the lower-tail shape of the force-variance distribution changes with speed. Under purely multiplicative or additive noise, all quantile curves would share one exponent.

**Table 3:** Sensitivity of the discovery to the quantile level τ.

τ	Tracking: selected family	Pushing: selected family	Pushing α [95% CI]
0.005	saturation	power law	2.41 [2.18, 2.51]
0.01	saturation	power law	2.17 [2.07, 2.24]
0.02	saturation	power law	2.02 [1.88, 2.17]
0.05	saturation	power law	1.46 [1.25, 1.72]
0.10	logarithmic (tie, + 0.04)	linear (tie, + 0.16)	n/a (power law not selected)

### 6.8. Robustness across trajectory geometries

Because the tracking design crosses speed with geometry, the discovery can be repeated within each geometry separately. The saturation family wins independently in all three, at square (ΔBIC to runner-up +11.96, $R^{\text{2}}=\text{0}.\text{9}\text{6}\text{7}$), figure-eight (+7.21, $R^{\text{2}}=\text{0}.\text{9}\text{6}\text{6}$), and circle (+2.43, $R^{\text{2}}=\text{0}.\text{9}\text{1}\text{5}$), with geometry-specific asymptotes of approximately 7.6, 11.8, and 6.4 mm respectively, each fitted to boundary points that pool the three repetitions of every condition. The functional form is invariant while the level is geometry-dependent, since higher-curvature paths saturate at higher error. This sharpens the interpretation of the pooled boundary as a statement about the task family, with the pooled $E_{max}$ a mixture-weighted compromise among geometry-specific asymptotes. The prospective validation of Section 6.11 tests exactly this decomposition on unseen geometries and unseen speeds.

### 6.9. Synthetic ground-truth validation

The theoretical guarantees of Section 4 are tested directly on synthetic data with known boundaries. For each of the five families as ground truth, data are generated as the true boundary plus exponential noise (σ=3 mm), and the full pipeline is run over a grid of bin counts B∈{4,…,16} and per-bin sizes $n_{b}\in \{\text{5}\text{0},\ldots ,\text{2}\text{0}\text{0}\text{0}\}$ (50 replicates per cell; Fig 6). For curvature-separated truths, saturation and power law, the families discovered in both real datasets, selection accuracy is 100% at every configuration, including well below the experimental B=8–10 and $n_{\text{min}}\geq \text{1},\text{0}\text{8}\text{9}$. For near-degenerate truths (linear, logarithmic, square root), which are nearly indistinguishable over short input ranges, accuracy is limited at small B (19–29% at B=4) and improves with B (to 75–84% at B=16), exactly the regime that small reported ΔBIC margins flag. Under a misspecified truth outside the library (a saturating base plus linear drift), the projection-optimal family predicted by Proposition 1 is selected in 100% of replicates and the fitted curve tracks the true τ-quantile curve to within a root-mean-square error of 0.06 mm. The estimator projects onto the library instead of failing unpredictably. A companion experiment addresses the nested case of Remark 2: with a linear ground truth, which the power law contains as the special case of unit exponent, BIC prefers the smaller family in roughly three quarters of replicates, and this frequency does not increase with the per-bin sample size, confirming that parsimony is not selected consistently at fixed B.

**Fig 6:**
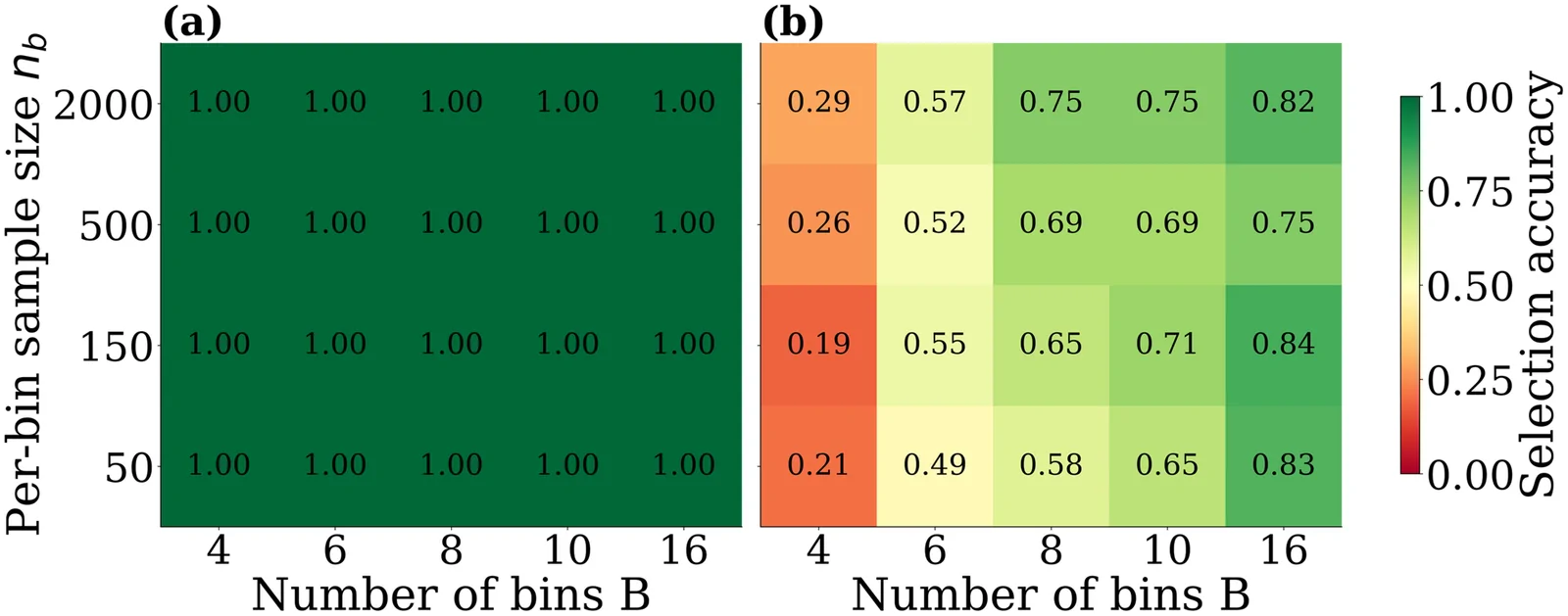
Synthetic ground-truth validation of family selection. (a) Accuracy for curvature-separated ground truths. (b) Accuracy for near-degenerate ground truths.

### 6.10. Extended baseline comparison and extrapolation

Beyond the machine-learning baselines of Table 2, Table 4 evaluates the estimators that are actually designed for lower envelopes. These are the FDH minimum, the order-α conditional frontier of Aragon, Daouia and Thomas-Agnan [32] at α=τ, the Cazals–Florens–Simar order-m expected-minimum frontier with m tuned to the 98% validity target, and gradient-boosted quantile regression (GBQR) [58] at τ=0.02. Two findings stand out. First, the true-minimum estimators exhibit exactly the noise fragility the Related Work anticipates. On tracking, a single near-zero transient error drags the entire FDH envelope to ~0.24 mm, producing strongly negative $R^{\text{2}}$ against the boundary points despite trivially perfect validity. Second, the nonparametric quantile methods (order-α, GBQR) match the empirical per-speed quantiles closely, unsurprisingly, since with discrete speed levels they essentially reproduce stage 2 of the pipeline, but deliver no functional form, no physical parameters, are not monotone on these data, and cannot extrapolate. The order-m frontier is the strongest competitor, with monotone and near-target validity, but it is looser than the proposed framework on both datasets.

**Table 4:** Frontier and quantile baselines on the boundary points (tracking dataset; pushing analogous, S2 Table).

Method	$R^{2}$	Validity	Tightness (mm)	Monotone	Functional form
Proposed (saturation)	0.962	0.921	0.32	yes	yes
FDH (minimum)	−4.71	1.000	4.29	yes	no
Order-α (α=0.02)	0.994	0.962	0.12	no	no
Order-m (98% target)	0.916	0.972	0.40	yes	no
GBQR (τ=0.02)	0.998	0.980	0.038	no	no

Extrapolation makes the parametric advantage quantitative. Fitting every method only on the low-speed bins and predicting the held-out high-speed boundary, the proposed framework is the only method that predicts the correct qualitative regime in both datasets. On tracking (truth plateaus at 7.1–7.7 mm) it predicts 7.8–8.0 mm, where a linear quantile fit overshoots to 12–15 mm (S2 Table). On pushing (truth rises to 1.54 N²) every flat-extending method predicts ~0.25 N², six-fold optimistic at 500 mm/s, while the framework predicts continued growth (0.80/1.86/3.61 N² at 300/400/500 mm/s against 0.61/0.94/1.54). For a lower envelope the error directions are not symmetric. Underpredicting the boundary promises performance the system cannot deliver, while overpredicting is conservative. The framework errs conservatively, and the step methods err optimistically. The complete extrapolation table is provided in S2 Table.

### 6.11. Prospective validation and reproducibility

The strongest test of a discovered law is prediction. Five quantitative predictions were therefore specified and recorded in advance of the validation experiments (S1 File), computed solely from the boundaries fitted to the main experimental campaign: (P1) at least 95% of all new observations lie above the pooled boundary; (P2) the 2nd-percentile error, pooled over the three training geometries, at the never-tested speeds 0.35 and 0.40 m/s remains within [6.5, 9.0] mm, whereas linear extrapolation of the low-speed slope predicts above 12 mm; (P3) per-geometry point predictions at both new speeds from the geometry-specific saturation laws; (P4) three novel geometries never present in training (star, spiral, smoothed random waypoints) select a saturating family; and (P5) their asymptotes fall within the training range [5.5, 12] mm. The predictions were computed from the fitted boundaries, written to a timestamped file, and deposited unchanged in the public repository (S1 File); no validation data existed at that point. The session comprised 72 new episodes (36 novel-geometry episodes at four in-range speeds; 36 extrapolation episodes across all six geometries at 0.35 and 0.40 m/s). Table 5 reports the scorecard, and Fig 7 visualizes it. Panel (a) plots the six frozen point predictions against their outcomes, and panel (b) plots the boundaries discovered for the novel geometries. P1 passed (95.7% of 21,852 observations). P2 passed at both speeds (8.28 and 8.90 mm). P3 produced six-for-six predictions within 1.2 mm, and the circle boundary predicted the unseen-speed 2nd percentile to within 0.07 and 0.01 mm. P4 and P5 each scored two of three, with both misses concentrated in the smoothed-random geometry. Its lower curvature demand places its boundary (~3.8 mm asymptote) below the training family’s range, and its small dynamic range (2.2–3.2 mm over the tested speeds) is precisely the near-degenerate regime that the synthetic study of Section 6.9 identifies as unidentifiable, flagged, as recommended, by a ΔBIC margin of 0.3. The refined claim these results support is that the functional form and the asymptote-governed regime transfer to unseen geometries and unseen speeds, while the level is conditional on the task family.

**Table 5:** Prospective prediction scorecard. Predictions were specified and recorded in advance of the validation experiments (S1 File).

Prediction	Result	Verdict
P1: ≥ 95% of new observations above the pooled boundary	95.7% (n = 21,852)	pass
P2: 2%ile over the three training geometries at 0.35/ 0.40 m/s within [6.5, 9.0] mm	8.28/ 8.90 mm	pass
P3: per-geometry point predictions (6 values)	misses +0.76/ +1.12 (square), −0.07/ +0.01 (circle), +0.32/ +0.85 mm (figure-8)	6/6 within 1.2 mm
P4: novel geometries select a saturating family	star (+4.1), spiral (+6.8); random: no reliable family (ΔBIC 0.3)	2/3
P5: novel asymptotes within [5.5, 12] mm	star 7.6, spiral 6.8; random ~3.8	2/3

**Fig 7:**
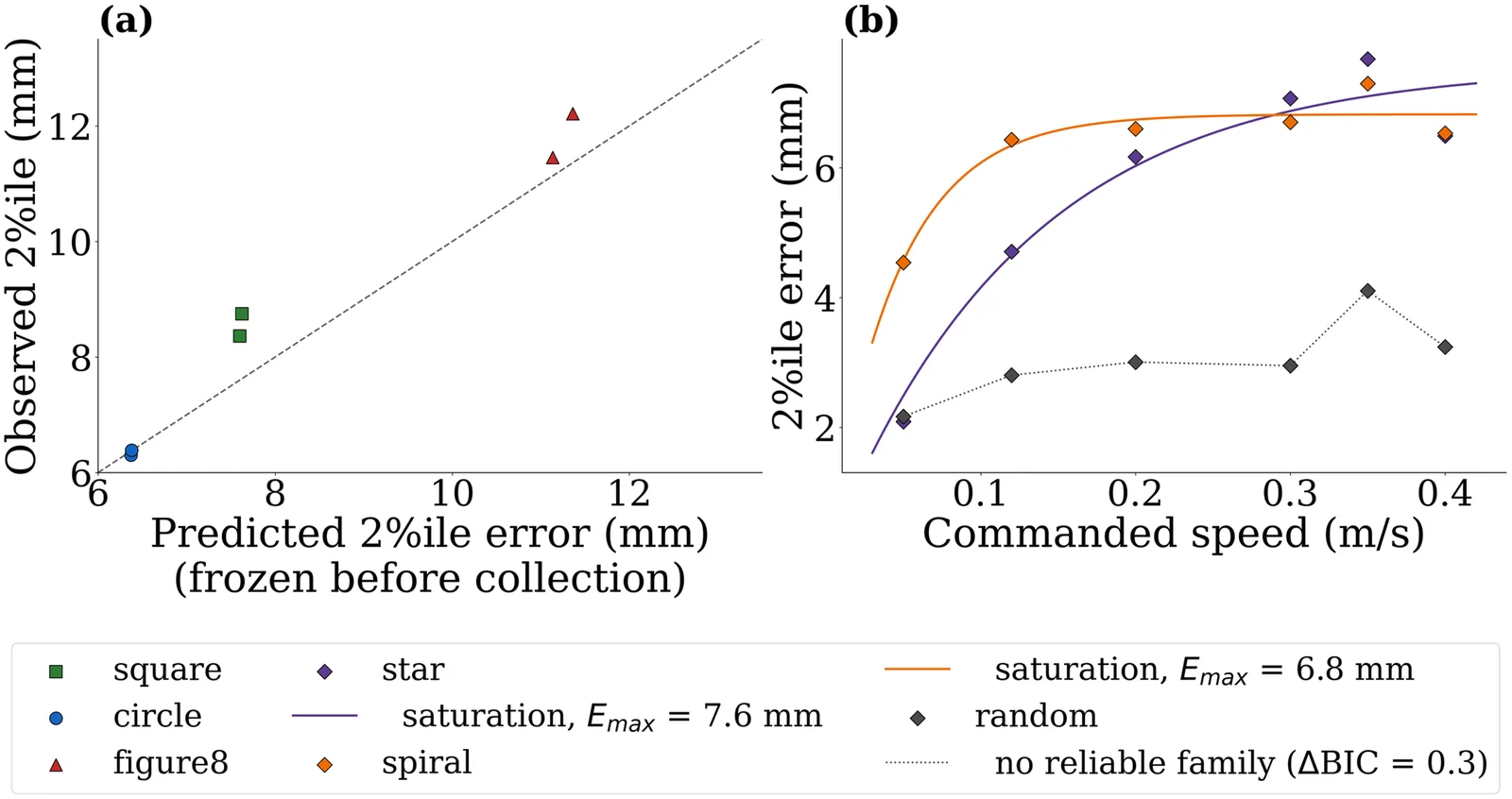
Prospective validation of the discovered boundaries. (a) Predicted versus observed extrapolation to never-fitted speeds. (b) Boundaries discovered for novel geometries.

Nine conditions of the main protocol were additionally repeated (three geometries at three speeds). Table 6 compares these values with the corresponding per-episode 2nd percentiles from the main experiments. These per-episode values are systematically higher than the boundary points of Section 6.2. The difference is an interpolation effect. Using the linear-interpolation rule of the usual type-7 quantile definition, a high-speed episode of 131 samples places the 2nd percentile between the third and fourth order statistics, whereas pooling the three square repetitions at that speed, 393 samples in total, places it essentially on the third smallest value. On the steep start-up ramp those values differ by more than a millimeter, which is why a single square episode at 0.30 m/s gives 8.41 mm while the square-only pooled value is 7.09 mm, even though the repetitions agree with one another to hundredths of a millimeter. Boundary points at the highest speeds therefore depend on how many episodes are pooled. The episode bootstrap of Section 6.6 inherits this sensitivity, since resampling with replacement duplicates episodes and changes the pooled sample in the same way; the resulting variation is part of what the reported intervals measure rather than an artifact they remove. Because the prospectively specified predictions and their outcomes were computed with the same pooling and the same quantile rule, the comparison is internally consistent, but the 1.2 mm tolerance of P3 should be read against a boundary-point definition that carries a sensitivity of this size. The mean absolute deviation across the nine conditions is 0.039 mm, the maximum 0.098 mm, and the correlation 0.9999, so the per-episode 2nd percentiles reproduce across independent runs of the protocol, including a robot re-jog and an unrecorded tool orientation.

**Table 6:** Reproducibility of the per-episode 2nd percentile of tracking error. Nine geometry–speed conditions, main experiments versus repeat protocol. Values in mm.

Condition	Primary experiments, mean [range]	Repeat	Deviation
square @ 0.05	3.63 [3.60, 3.66]	3.69	+0.062
square @ 0.12	7.11 [7.10, 7.11]	7.05	−0.055
square @ 0.30	8.41 [8.41, 8.42]	8.42	+0.002
circle @ 0.05	4.23 [4.22, 4.24]	4.24	+0.016
circle @ 0.12	6.09 [6.07, 6.13]	6.19	+0.098
circle @ 0.30	7.13 [7.11, 7.16]	7.16	+0.030
figure-8 @ 0.05	3.32 [3.31, 3.32]	3.35	+0.035
figure-8 @ 0.12	7.88 [7.87, 7.89]	7.88	+0.000
figure-8 @ 0.30	11.55 [11.53, 11.56]	11.60	+0.055

### 6.12. Summary of discovered boundaries

Table 7 summarizes the discovered boundaries. Both yield closed-form equations with physically interpretable parameters, both fit the boundary points with high fidelity, and both expose mechanisms, the transient-regime envelope with its steady-state lag-law counterpart (Section 6.5), and momentum-transfer scaling, that linear models would miss entirely. Fig 8 compares the two boundaries on normalized axes to make the opposite regimes directly visible. The tracking boundary is concave (diminishing returns), and the pushing boundary is convex (accelerating difficulty). Boundary points are normalized by their per-dataset minimum and maximum observed values, and fitted curves by their range over the fitted interval, so the points and the curves are placed on comparable but not identical scales.

**Table 7:** Summary of discovered boundaries.

Domain	Selected family	Parameters	$R^{2}$	Physical interpretation
Robot tracking	Saturation	$E_{\text{max}}=\text{7}.\text{7}\text{4}$, k=13.27 ($E_{\text{max}}$ in mm, k in s/m)	0.962	Transient-regime envelope; steady-state lag $t_{lag}$ = 65 ms (Sec. 6.5)
Object pushing	Power law	$k=\text{5}.\text{3}\text{6}\times {\text{1}\text{0}}^{-\text{6}}$, α=2.02, $a\;=\;\text{0}.\text{0}\text{1}\text{2}\;{\text{N}}^{\text{2}}$ (k in N²·(mm/s)^-2.02^)	0.997	Momentum-transfer scaling

**Fig 8:**
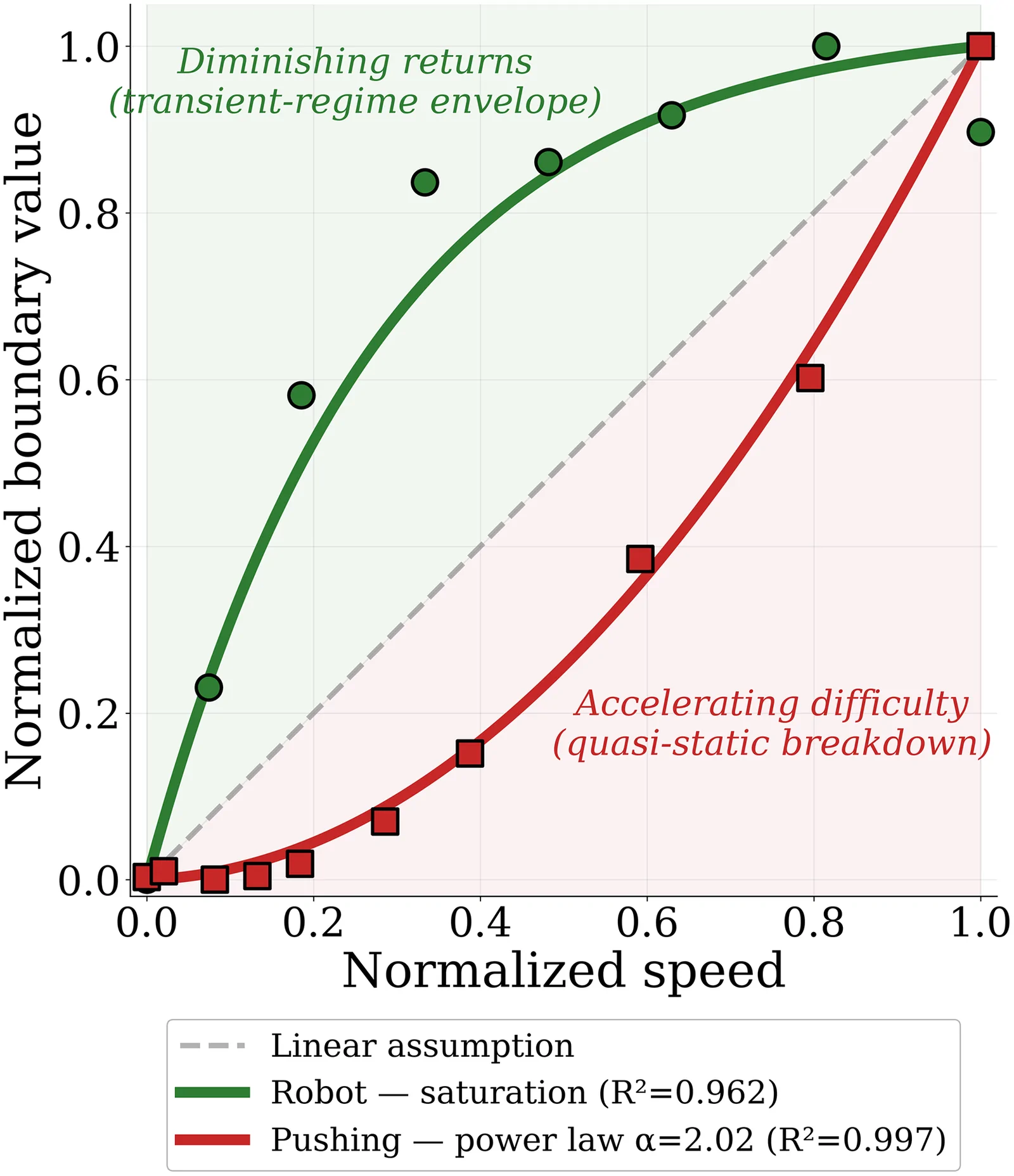
Normalized comparison of the two discovered boundaries on unit axes.

## 7. Conclusion

This paper developed a quantile-based framework for discovering performance boundaries in physical systems, combining a binned percentile estimator with parametric model selection by BIC across a library of physically motivated families, supported by non-asymptotic theory stated in the fixed-design regime the experiments occupy. On a UR10e tracking dataset, the framework discovers a saturation boundary $E=\text{7}.\text{7}\text{4}\left(\text{1}-e^{-\text{1}\text{3}.\text{2}\text{7}S}\right)$ mm ($R^{\text{2}}=\text{0}.\text{9}\text{6}\text{2}$; $E_{max}$ 95% CI [7.05,9.10] mm) for the process as recorded, and separates it from a steady-state envelope following the lag law $e=t_{lag}v$ with $t_{lag}=\text{6}\text{5}$ ms ($R^{\text{2}}=\text{0}.\text{9}\text{9}\text{7}$) once start-up transients are excluded, a two-regime structure that the framework itself surfaced, whose recorded-process envelope was confirmed by prospectively specified predictions at never-tested speeds and whose steady-state envelope was confirmed by a post hoc comparison at the same speeds. On the public MIT MCube pushing dataset, the framework discovers a power-law boundary with exponent α=2.02 (95% CI [1.88,2.17], containing the momentum-transfer value 2), robust to push-level and shape-cluster resampling. Family selection is stable across quantile levels τ∈[0.005,0.05] on both datasets, verified against synthetic ground truths where the discovered family classes are identified with 100% accuracy at the experimental configurations, and validated by a prospectively specified experiment in which six of six extrapolation point predictions landed within 1.2 mm (two within 0.1 mm) and a repeat of the protocol reproduced the per-episode 2nd percentiles to a mean of 0.039 mm.

The framework has limitations that motivate future work. First, the bin grid is determined by the experimental design; adaptive selection of test conditions to shrink boundary uncertainty fastest, active boundary discovery, is a natural extension that the fixed-design theory directly supports. Second, the candidate library is rich but not exhaustive; under misspecification the framework provably and empirically selects the projection-optimal family, but multi-scale phenomena may warrant richer libraries. Third, estimation is at a fixed extreme level τ; extrapolation to the τ→0 limit via extreme-value theory [8] would connect the estimated envelope to the essential-infimum boundary of Definition 2 in cases where that limit is non-degenerate. Finally, the two-regime finding carries a general caution and an opportunity. What counts as an observation is part of the estimand, and applying the framework under several observation definitions, as done here for transient-inclusive and steady-state tracking, turns that sensitivity into a source of physical insight rather than a threat to validity.

## Supporting information

S1 Appendix Proofs of Theorem 2, Theorem 3, Proposition 1, and the clustered variant of Lemma 1. (DOCX)

S1 File Prospectively specified predictions. Timestamped record of the five predictions of Section 6.11, written before the validation experiments were carried out.
(JSON)

S1 Table Per-bin boundary points with uncertainty certificates. Boundary points, bootstrap confidence intervals, and finite-sample certificates for both datasets.
(DOCX)

S2 Table Frontier-baseline comparison and extrapolation benchmark. Comparison with frontier and quantile baselines on both datasets, and the extrapolation benchmark.
(DOCX)
