# Supplementary material for: Data-driven discovery of performance boundaries in robotic systems through extreme quantile estimation
Source: PLoS One. 2026 Nov 10;21(11):e0352196. doi: 10.1371/journal.pone.0352196 (PMC13750611; doi:10.1371/journal.pone.0352196)
Supplement: Supplementary file 1 [file pone.0352196.s001.docx]

**S1 Appendix. Proofs of Theorem 2, Theorem 3 and Proposition 1, and the clustered variant of Lemma 1.**

**A.1. Proof of Theorem 2(a)**

Write $Q\left( \theta\right)=\sum_{b} \left( f_{\tau}^{*}\left( x_{b} \right)-f\left( x_{b};\theta\right) \right)^{2}$ for the population criterion and $\hat{Q}\left( \theta\right)=\sum_{b} \left( \hat{q}_{\tau,b}-f\left( x_{b};\theta\right) \right)^{2}$ for the empirical one, and abbreviate $e_{b}=\hat{q}_{\tau,b}-f_{\tau}^{*}\left( x_{b} \right)$, so that on the event of Lemma 1 of the main text, $\max_{b}\left| e_{b} \right|\leq\varepsilon$. Expanding each summand, for every $\theta\in\Theta$,

$$|\hat{Q}\left( \theta\right)-Q\left( \theta\right)|\leq\sum_{b} \left( 2\left| e_{b} \right| \left| f_{\tau}^{*}\left( x_{b} \right)-f\left( x_{b};\theta\right) \right|+e_{b}^{2} \right)\leq B\varepsilon\left( 2M+\varepsilon\right)=:\Delta.$$

Since $\hat{\theta}$ minimizes $\hat{Q}$ and $Q\left( \theta^{*} \right)=0$,

$$Q\left( \hat{\theta} \right)\leq\hat{Q}\left( \hat{\theta} \right)+\Delta\leq\hat{Q}\left( \theta^{*} \right)+\Delta\leq Q\left( \theta^{*} \right)+2\Delta=2\Delta.$$

By Assumption 3, a second-order Taylor expansion of $\theta\mapsto\left( f\left( x_{1};\theta\right),\ldots,f\left( x_{B};\theta\right) \right)$ at $\theta^{*}$ gives $Q\left( \theta\right)=\left( \theta-\theta^{*} \right)^{\top}G^{\top}G \left( \theta-\theta^{*} \right)+R\left( \theta\right)$ with $\left| R\left( \theta\right) \right|\leq C_{2}\parallel\theta-\theta^{*}\parallel^{3}$ for a curvature constant $C_{2}$ on the compact $\Theta$. Choose $r_{0}=\kappa/\left( 2C_{2} \right)$ (if $C_{2}=0$, any $r_{0}$); then for $\parallel\theta-\theta^{*}\parallel\leq r_{0}$, $Q\left( \theta\right)\geq\kappa/2\parallel\theta-\theta^{*}\parallel^{2}$ (quadratic growth), and by Assumption 4 together with compactness, $q_{0}:=\inf_{\parallel\theta-\theta^{*}\parallel>r_{0}}Q\left( \theta\right)>0$ (the infimum of a continuous positive function over a compact set bounded away from the unique root). If $2\Delta<q_{0}$, then $Q\left( \hat{\theta} \right)\leq2\Delta<q_{0}$ forces $\parallel\hat{\theta}-\theta^{*}\parallel\leq r_{0}$, whence $\kappa/2\parallel\hat{\theta}-\theta^{*}\parallel^{2}\leq2\Delta$, i.e. $\parallel\hat{\theta}-\theta^{*}\parallel\leq2\sqrt{\Delta/\kappa}$, Eq (12) of the main text. $\square$

**A.2. Proof of Theorem 2(b)**

Under Assumptions 2 and 5, the per-bin empirical quantiles satisfy, independently across bins, $\sqrt{n_{b}} \left( \hat{q}_{\tau,b}-f_{\tau}^{*}\left( x_{b} \right) \right)\mathcal{\Rightarrow N}\left( 0,s_{b}^{2} \right)$ with $s_{b}^{2}=\tau\left( 1-\tau\right)/p_{b}\left( f_{\tau}^{*}\left( x_{b} \right) \right)^{2}$ ([A1], Chapter 21, Corollary 21.5). Stacking, $\sqrt{n_{\min}} \left( \hat{q}_{\tau}-f_{\tau}^{*} \right)\mathcal{\Rightarrow N}\left( 0,S \right)$ with $S$ as in Eq (14) of the main text, using $n_{b}/n\to w_{b}$ so that $n_{\min}/n_{b}$ converges for every $b$. By Theorem 2(a) and Lemma 1, $\hat{\theta}\to\theta^{*}$ in probability; on a neighborhood of $\theta^{*}$ the map $T$ sending a $B$-vector $q$ to the minimizer of $\sum_{b} \left( q_{b}-f\left( x_{b};\theta\right) \right)^{2}$ is well defined and continuously differentiable by the implicit function theorem applied to the score equation $\sum_{b} \left( q_{b}-f\left( x_{b};\theta\right) \right) \nabla_{\theta}f\left( x_{b};\theta\right)=0$, whose $\theta$-derivative at $\left( f_{\tau}^{*},\theta^{*} \right)$ is $-G^{\top}G$ (invertible by Assumption 4) and whose $q$-derivative is $G^{\top}$. Hence $\nabla_{q}T\left( f_{\tau}^{*} \right)=\left( G^{\top}G \right)^{-1}G^{\top}$, and the delta method yields $\sqrt{n_{\min}} \left( \hat{\theta}-\theta^{*} \right)\mathcal{\Rightarrow N}\left( 0,\left( G^{\top}G \right)^{-1}G^{\top}SG\left( G^{\top}G \right)^{-1} \right)$, which is Eq (13) of the main text; see also [A2] for the fixed-design nonlinear least squares argument. $\square$

**A.3. Proofs of Theorem 3 and Proposition 1**

For each family $k$, let $Q_{k}\left( \theta\right)=\sum_{b} \left( f_{\tau}^{*}\left( x_{b} \right)-f_{k}\left( x_{b};\theta\right) \right)^{2}$ and $\hat{Q}_{k}\left( \theta\right)$ its empirical counterpart, so $\mathrm{RSS}_{k}=\min_{\theta}\hat{Q}_{k}\left( \theta\right)$ and $D_{k}=\min_{\theta}Q_{k}\left( \theta\right)$. The bound of A.1, applied verbatim to family $k$, gives $\left| \mathrm{RSS}_{k}-D_{k} \right|\leq\Delta_{k}:=B\varepsilon\left( 2M_{k}+\varepsilon\right)$ on the event of Lemma 1 of the main text, where $M_{k}$ is the envelope constant of family $k$; since $\varepsilon=\varepsilon\left( \eta\right)\to0$ as $n_{\min}\to\infty$ for every fixed $\eta$, it follows that $\mathrm{RSS}_{k}\to D_{k}$ in probability, for every $k$.

Proof of Theorem 3. For $k=k^{*}$, $D_{k^{*}}=0$ and the same computation gives $0\leq\mathrm{RSS}_{k^{*}}\leq\Delta_{k^{*}}=O_{P}\left( \varepsilon\right)$, hence $B\log\left( \mathrm{RSS}_{k^{*}}/B \right)\to-\infty$ in probability, which is Eq (17) of the main text. For $k\neq k^{*}$, $\mathrm{RSS}_{k}\to D_{k}>0$, so $B\log\left( \mathrm{RSS}_{k}/B \right)$ is bounded in probability. The penalties $p_{k}\log B$ are constants at fixed $B$. Therefore $\mathrm{BIC}_{k^{*}}-\mathrm{BIC}_{k}\to-\infty$ in probability for every $k\neq k^{*}$, and $\Pr\left( \hat{k}=k^{*} \right)\to1$. $\square$

Proof of Proposition 1. Now all $D_{k}>0$. For any $k$ with $D_{k}>D_{\tilde{k}}$, $\mathrm{BIC}_{k}-\mathrm{BIC}_{\tilde{k}}=B\log\left( \mathrm{RSS}_{k}/\mathrm{RSS}_{\tilde{k}} \right)+\left( p_{k}-p_{\tilde{k}} \right)\log B\to B\log\left( D_{k}/D_{\tilde{k}} \right)+\left( p_{k}-p_{\tilde{k}} \right)\log B$, and the first term is a strictly positive constant that can be made to dominate: more precisely $\mathrm{RSS}_{k}/\mathrm{RSS}_{\tilde{k}}\to D_{k}/D_{\tilde{k}}>1$ in probability, so $\mathrm{liminfPr}\left( \mathrm{BIC}_{k}>\mathrm{BIC}_{\tilde{k}} \right)\to1$ whenever $B\log\left( D_{k}/D_{\tilde{k}} \right)+\left( p_{k}-p_{\tilde{k}} \right)\log B>0$, which holds for all $B\geq2$ if $p_{k}\geq p_{\tilde{k}}$ and, for $p_{k}<p_{\tilde{k}}$, for all $B$ large enough; at fixed $B$ the statement is that the selection event has probability tending to one whenever the deterministic quantity $B\log\left( D_{k}/D_{\tilde{k}} \right)+\left( p_{k}-p_{\tilde{k}} \right)\log B$ is positive, a condition verified numerically for the fitted configurations and satisfied in every synthetic replicate. If instead $D_{k}=D_{\tilde{k}}$, then $B\log\left( \mathrm{RSS}_{k}/\mathrm{RSS}_{\tilde{k}} \right)=o_{P}\left( 1 \right)$ by the uniform convergence above, and the sign of $\mathrm{BIC}_{k}-\mathrm{BIC}_{\tilde{k}}$ is eventually that of $\left( p_{k}-p_{\tilde{k}} \right)\log B$, so the family with fewer parameters is selected with probability tending to one. Convergence of the fitted parameter to $\tilde{\theta}$ within the selected family is the standard M-estimation consistency argument: $\hat{Q}_{\tilde{k}}\to Q_{\tilde{k}}$ uniformly on $\Theta_{\tilde{k}}$ (the bound of A.1 is uniform in $\theta$), $Q_{\tilde{k}}$ has the unique minimizer $\tilde{\theta}$ by assumption, and $\Theta_{\tilde{k}}$ is compact. $\square$

**A.4. Clustered Observations: Lemma 1 with Effective Sample Size**

Suppose bin $b$ consists of $m_{b}$ independent episodes, episode $j$ contributing $n_{b,j}$ observations with weights $w_{j}=n_{b,j}/n_{b}$, $\sum_{j} w_{j}=1$, and that observations are identically distributed with marginal $F_{b}$ but arbitrarily dependent within an episode. The pooled empirical distribution function is $\hat{F}_{b}\left( y \right)=\sum_{j} w_{j}\hat{F}_{b,j}\left( y \right)$, where $\hat{F}_{b,j}$ is the empirical distribution function of episode $j$. Viewed as a function of the independent episode-level random elements, changing episode $j$ changes $\hat{F}_{b}\left( y \right)$ by at most $w_{j}$, uniformly in $y$; hence, for the supremum statistic $Z=\sup_{y}\left| \hat{F}_{b}\left( y \right)\mathbb{-E}\hat{F}_{b}\left( y \right) \right|$, McDiarmid’s inequality [A3] gives $\Pr\left( Z\mathbb{>E}Z+t \right)\leq exp\left( -2t^{2}/\sum_{j} w_{j}^{2} \right)$. Since $\mathbb{E}\hat{F}_{b}=F_{b}$ and $\mathbb{E}Z\to0$ as the number of episodes grows (by episode-level Glivenko–Cantelli), the quantity $n_{b}^{\mathrm{eff}}=1/\sum_{j} w_{j}^{2}$ plays the role of the sample size: with equal episode sizes, $n_{b}^{\mathrm{eff}}=m_{b}$. The quantile conversion step of the proof of Lemma 1 is unchanged, yielding, for $\varepsilon\leq\delta$ and $t=c_{b}\varepsilon\mathbb{-E}Z$ positive, $\Pr\left( \left| \hat{q}_{\tau,b}-f_{\tau}^{*}\left( x_{b} \right) \right|>\varepsilon\right)\leq exp\left( -2n_{b}^{\mathrm{eff}}\left( c_{b}\varepsilon\mathbb{-E}Z \right)^{2} \right)$, and a union bound over bins gives the clustered analogue of Eq (10) of the main text with $n_{b}$ replaced by $n_{b}^{\mathrm{eff}}$ up to the centering term $\mathbb{E}Z$. For the tracking dataset, $m_{b}=9$ episodes per bin with approximately equal sizes, so $n_{b}^{\mathrm{eff}}\approx9$ regardless of the thousands of recorded timesteps, the quantitative content of the statement, made in Section 4.5, that honest uncertainty is episode-limited in every bin. $\square$

**References**

A1.	Vaart AW van der. Asymptotic Statistics. Cambridge: Cambridge University Press; 1998. doi:10.1017/CBO9780511802256

A2.	Jennrich RI. Asymptotic Properties of Non-Linear Least Squares Estimators. The Annals of Mathematical Statistics. 1969;40: 633–643.

A3.	McDiarmid C. On the method of bounded differences. In: Siemons J, editor. Surveys in Combinatorics, 1989: Invited Papers at the Twelfth British Combinatorial Conference. Cambridge: Cambridge University Press; 1989. pp. 148–188. doi:10.1017/CBO9781107359949.008
