# Supplementary material for: Data-driven discovery of performance boundaries in robotic systems through extreme quantile estimation
Source: PLoS One. 2026 Nov 10;21(11):e0352196. doi: 10.1371/journal.pone.0352196 (PMC13750611; doi:10.1371/journal.pone.0352196)
Supplement: Supplementary file 2 [file pone.0352196.s003.docx]

**S1 Table. Per-bin boundary points with uncertainty certificates.**

Per-bin 2nd-percentile boundary points with bootstrap 95% confidence intervals and worst-case Dvoretzky-Kiefer-Wolfowitz (DKW) certificates. Confidence intervals are computed by an episode-level bootstrap for the tracking dataset (1,500 replicates resampling the nine episodes within each commanded speed) and by a push-level bootstrap for the pushing dataset (800 replicates). DKW certificates follow Lemma 1 of the main text, evaluated with kernel plug-in estimates of the tail density *c*_b_ and confidence parameter *η* = 0.05 allocated across bins. The DKW envelopes are worst-case certificates. They are informative where the lower tail is steep, at low speeds, and become uninformative where it is flat. At the highest tracking speeds the certified neighborhood would extend below zero error, where the density vanishes and Assumption 2 of the main text no longer holds, so the envelopes in the last rows of Table A are reported for completeness only. The bootstrap intervals are the calibrated uncertainty used for inference. All quantiles are computed with the linear-interpolation (type-7) rule; Section 6.6 of the main text reports the sensitivity of the results to this choice.

**Table A in S1 Table. Robot tracking dataset (values in mm).**

| **Speed (m/s)** | **Boundary point *q*_τ_** | **Bootstrap 95% CI** | **Tail density *c*_b_ (mm^−1^)** | **DKW envelope ±** |
| --- | --- | --- | --- | --- |
| 0.03 | 2.034 | [2.005, 2.131] | 0.260 | 0.062 |
| 0.05 | 3.332 | [3.320, 3.544] | 0.134 | 0.156 |
| 0.08 | 5.307 | [5.283, 5.408] | 0.078 | 0.337 |
| 0.12 | 6.746 | [6.068, 7.362] | 0.0047 | 6.86 |
| 0.16 | 6.883 | [5.967, 8.103] | 0.0038 | 9.73 |
| 0.20 | 7.198 | [6.151, 8.552] | 0.0036 | 11.81 |
| 0.25 | 7.665 | [5.876, 8.187] | 0.0035 | 13.45 |
| 0.30 | 7.086 | [7.032, 9.296] | 0.0034 | 15.33 |

**Table B in S1 Table. Planar pushing dataset (values in N**^2^**).**

| **Speed (mm/s)** | **Boundary point *q*_τ_** | **Bootstrap 95% CI** | **DKW envelope ±** |
| --- | --- | --- | --- |
| 10 | 0.0268 | [0.0146, 0.0363] | 0.055 |
| 20 | 0.0373 | [0.0025, 0.0618] | 0.085 |
| 50 | 0.0219 | [0.0111, 0.0750] | 0.213 |
| 75 | 0.0286 | [0.0257, 0.0699] | 0.304 |
| 100 | 0.0512 | [0.0483, 0.0766] | 0.376 |
| 150 | 0.1281 | [0.1208, 0.1401] | 0.459 |
| 200 | 0.2520 | [0.2335, 0.3368] | 0.525 |
| 300 | 0.6074 | [0.5706, 0.6623] | 0.676 |
| 400 | 0.9371 | [0.9052, 0.9962] | 0.852 |
| 500 | 1.5428 | [1.5044, 1.6045] | 1.025 |
