# Supplementary material for: Data-driven discovery of performance boundaries in robotic systems through extreme quantile estimation
Source: PLoS One. 2026 Nov 10;21(11):e0352196. doi: 10.1371/journal.pone.0352196 (PMC13750611; doi:10.1371/journal.pone.0352196)
Supplement: Supplementary file 3 [file pone.0352196.s004.docx]

**S2 Table. Frontier-baseline comparison and extrapolation benchmark.**

Comparison of the proposed framework with estimators designed for lower envelopes, on both datasets. Metrics are defined as in Table 2 of the main text. FDH denotes the free-disposal-hull minimum; Order-*α* denotes the conditional order-*α* frontier evaluated at *α* = *τ* = 0.02; Order-*m* denotes the Cazals-Florens-Simar expected-minimum frontier with *m* tuned toward the 98% validity target (*m* = 40 for tracking, *m* = 160 for pushing); GBQR denotes gradient-boosted quantile regression at *τ* = 0.02.

**Table A in S2 Table. In-range comparison on the boundary points, robot tracking dataset.**

| **Method** | ***R*^2^** | **Validity** | **Tightness (mm)** | **Monotone** | **Closed-form law** |
| --- | --- | --- | --- | --- | --- |
| Proposed (saturation) | 0.962 | 0.921 | 0.317 | yes | yes |
| FDH (minimum) | −4.71 | 1.000 | 4.293 | yes | no |
| Order-*α* | 0.994 | 0.962 | 0.120 | no | no |
| Order-*m* (*m* = 40) | 0.916 | 0.972 | 0.402 | yes | no |
| GBQR | 0.998 | 0.980 | 0.038 | no | no |

**Table B in S2 Table. In-range comparison on the boundary points, planar pushing dataset.**

| **Method** | ***R*^2^** | **Validity** | **Tightness (N^2^)** | **Monotone** | **Closed-form law** |
| --- | --- | --- | --- | --- | --- |
| Proposed (power law) | 0.997 | 0.980 | 0.0205 | yes | yes |
| FDH (minimum) | 0.844 | 1.000 | 0.1422 | yes | no |
| Order-*α* | 0.682 | 0.953 | 0.2374 | yes | no |
| Order-*m* (*m* = 160) | 0.982 | 0.981 | 0.0377 | yes | no |
| GBQR | 1.000 | 0.980 | 0.0018 | no | no |

In the extrapolation benchmark, each method is fitted only on the low-speed bins (six bins up to 0.20 m/s for tracking; seven bins up to 200 mm/s for pushing) and then predicts the held-out high-speed boundary points. Flat extension denotes the last training-bin quantile carried forward, which is the behavior of FDH, Order-*α*, and GBQR beyond the training range. Linear QR denotes a linear quantile fit (pinball loss, *τ* = 0.02) to the raw low-speed observations.

**Table C in S2 Table. Extrapolation to held-out speeds, robot tracking dataset (mm).**

| **Method** | **Prediction at 0.25 m/s** | **Prediction at 0.30 m/s** |
| --- | --- | --- |
| Held-out boundary point | 7.665 | 7.086 |
| Proposed (saturation) | 7.81 | 8.01 |
| Flat extension | 7.20 | 7.20 |
| Linear QR | 12.24 | 14.56 |

**Table D in S2 Table. Extrapolation to held-out speeds, planar pushing dataset (N**^2^**).**

| **Method** | **At 300 mm/s** | **At 400 mm/s** | **At 500 mm/s** |
| --- | --- | --- | --- |
| Held-out boundary point | 0.607 | 0.937 | 1.543 |
| Proposed (power law) | 0.80 | 1.86 | 3.61 |
| Flat extension | 0.25 | 0.25 | 0.25 |
| Linear QR | 0.31 | 0.41 | 0.51 |

The proposed framework is the only method that predicts the correct qualitative regime on both datasets. It reproduces the plateau on tracking, where Linear QR overshoots by roughly a factor of two, and the continued growth on pushing, where flat extension and Linear QR are three- to six-fold optimistic at 500 mm/s. For a lower envelope the two error directions are not equivalent, because underprediction promises performance that the system cannot deliver whereas overprediction is conservative. The framework errs conservatively on both datasets.
